# Gut Microbiota Alterations Associated With Colonization by Multidrug‐Resistant Organisms in ICU Patients: First Systematic Review and Meta‐Analysis

**DOI:** 10.1002/mbo3.70322

**Published:** 2026-06-21

**Authors:** Andra‐Elena Goicea, Daniel‐Corneliu Leucuța, Constantin Bodolea

**Affiliations:** ^1^ Department of Anesthesia and Intensive Care “Iuliu Hațieganu” University of Medicine and Pharmacy Cluj‐Napoca Romania; ^2^ Department of Anesthesia and Intensive Care Emergency Military Clinical Hospital “Dr. Constantin Papilian” Cluj‐Napoca Romania; ^3^ Department of Medical Informatics and Biostatistics “Iuliu Hațieganu” University of Medicine and Pharmacy Cluj‐Napoca Romania; ^4^ Anesthesia and Intensive Care Unit Municipal Clinical Hospital Cluj‐Napoca Romania

**Keywords:** bacterial colonization, critical care, dysbiosis, gastrointestinal microbiota, multidrug‐resistant bacteria

## Abstract

Colonization by multidrug‐resistant organisms (MDROs) is a risk factor for infection and mortality in critically ill patients, yet current strategies to prevent or control dissemination show variable effectiveness. Whether alterations in microbiota structure and composition (dysbiosis) are associated with MDRO colonization in critically ill patients is an open question this study aims to address. We conducted the first systematic review and meta‐analysis comparing intestinal microbiota structure (diversity) and composition (relative abundance of bacteria) between colonized critically ill patients and noncolonized/control patients. PubMed, Web of Science, and Scopus were systematically searched from inception to September 2025. The study protocol was preregistered on Open Science Framework, under embargo. Of 3003 records identified, 11 studies (*n* = 2823 patients) published between 2019 and 2025 met the inclusion criteria. The most frequently reported colonizing strains in MDROs‐colonized patients were vancomycin‐resistant Enterococci (58.2%), carbapenem‐resistant Enterobacterales (21.6%), extended‐spectrum β‐lactamase–producing Enterobacterales (13.4%). All studies employed 16S ribosomal RNA sequencing to assess intestinal microbiota. Colonized critically ill patients had lower values of dominance/evenness and richness, reaching significantly lower values of information when compared with controls (mean difference in Shannon index = –1.18; 95% CI, –1.84 to –0.52; *p* < 0.001). Composition investigation revealed a significant increase in the Pseudomonadota phylum and the Enterobacteriaceae family in MDROs‐colonized patients. Current evidence is limited and largely associational. Nevertheless, altered intestinal microbiota consistently characterize colonized critically ill patients. Further studies are warranted to determine whether early detection of dysbiosis can enhance early prediction/diagnosis of MDROs‐colonization and guide the targeted microbiota‐based strategies to prevent infections.

## Graphical abstract

Microbiota profiling showed colonized critically ill patients had lower values of dominance/evenness (Simpson's index) and richness (Observed), reaching significantly lower values of information (mean difference in Shannon index = –1.18; 95% CI, –1.84 to –0.52; *p* < 0.001).

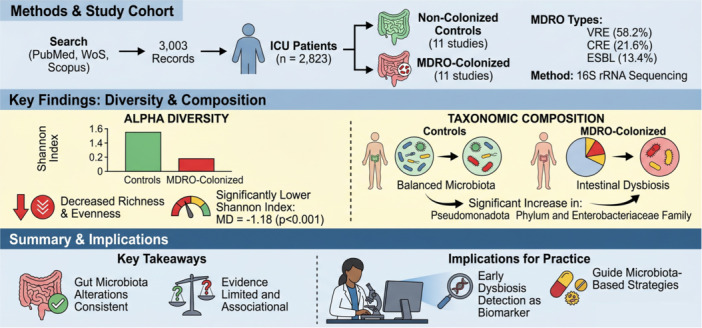

**Abbreviations**
AUC: area under the curve
CI: confidence interval
CRE: carbapenem‐resistant Enterobacterales
CRPA: carbapenem‐resistant *Pseudomonas aeruginosa*
ESBL‐E: extended‐spectrum β‐lactamase–producing Enterobacterales
*I* ^2^
: percentage of variance attributable to study heterogeneity
ICU: intensive care unit
inverse Simpson index: invSimpson index
MD: mean difference
MDROs: multidrug‐resistant organisms
MRSA: methicillin‐resistant *Staphylococcus aureus*
OTU: operational taxonomic unit
PCR: polymerase chain reaction
PRISMA: preferred reporting items for systematic reviews and meta‐analyses
rRNA: ribosomal ribonucleic acid
SD: standard deviation
STORMS: Strengthening the Organization and Reporting of Microbiome Studies
VRE: vancomycin‐resistant Enterococci

## 1 Introduction

Antimicrobial resistance remains a major global public health concern, with infections caused by multidrug‐resistant organisms (MDROs) presenting ongoing challenges to healthcare systems (CDC 2025; Organization 2025). Resistance to “Watch” antibiotics is increasing among key Gram‐negative species (CDC 2025). Estimates suggest that morbidity, mortality, and healthcare costs associated with MDROs will rise substantially worldwide by 2050 (Naghavi et al. 2024).

Colonization with MDROs is of particular concern, as it is associated with an overall incidence of secondary infections with MDROs of 22%, and it happens as early as 4 days after admission to the intensive care unit (ICU), the relationship being almost linear between time in ICU and colonization (Heath et al. 2024; Woodhouse et al. 2025). While there is no universally accepted definition for a normal intestinal microbiota given the broad range of host and environmental factors related to it, its major role in colonization resistance through the microbial diversity, competition for resources and niches, production of metabolites, such as short‐chain fatty acids (SCFAs), and maintenance of intestinal barrier integrity, is well recognized (Berg et al. 2020; Hou et al. 2022; Van Hul et al. 2024).

Advances in next‐generation sequencing have improved microbiota characterization through 16S ribosomal ribonucleic acid (rRNA) gene sequencing, shotgun metagenomics, and RNA sequencing (Wensel et al. 2022). Microbiota is generally described in terms of structure, measured as α‐diversity (within‐sample diversity) and β‐diversity (between‐community similarity), and in terms of composition, assessed based on the relative abundance of bacterial taxa, which may influence host susceptibility or resilience (Su 2021; Van Hul et al. 2024). Cassol et al. (2025) recently proposed a standardized framework for α‐diversity metrics, grouping them into four categories: information, dominance/evenness, richness, and phylogenetic diversity.

Critically ill patients are a population of particular relevance because they are exposed to environmental and therapeutic conditions (antibiotic exposure, vasoactive therapy, artificial nutrition, renal replacement therapy, and mechanical ventilation) that favor the transition of commensal opportunistic bacteria toward pathogenicity (Zaborin et al. 2014; Neag et al. 2025).

In this context, we questioned whether ICU patients colonized with MDROs exhibit specific alterations in intestinal microbiota structure and composition. This systematic review and meta‐analysis aim to assess and synthesize the available evidence on this association. The primary outcome is to provide a comprehensive overview of alterations in microbiota structure and composition in critically ill patients colonized with MDROs. The secondary outcome is to integrate current evidence on microbiota changes in patients colonized explicitly with vancomycin‐resistant *Enterococcus* (VRE), extended‐spectrum β‐lactamase–producing Enterobacterales (ESBL‐E), and carbapenem‐resistant Enterobacterales (CRE).

## 2 Materials and Methods

The systematic review and meta‐analysis are reported in accordance with the 2020 Preferred Reporting Items for Systematic Reviews and Meta‐Analyses (PRISMA) guidelines (Page et al. 2021). The study protocol was preregistered on the Open Science Framework in December 2025 and is embargoed.

### 2.1 Eligibility Criteria

We included case–control, cross‐sectional, and cohort studies of adult ICU patients screened for MDROs, ESBL‐E, CRE, or VRE via rectal swabs or stool, with intestinal microbiota analyzed using molecular sequencing. We excluded studies of neonatal ICU patients, ICU patients screened for methicillin‐resistant *Staphylococcus aureus* (MRSA), studies assessing MDROs‐colonization without microbiota analysis, and nonoriginal research (case reports, editorials, guidelines, letters, or reviews).

### 2.2 Research Strategy and Data Screening

A literature search was conducted in September 2025 across PubMed, Web of Science, and Scopus, with no language or publication‐year restrictions. Search queries were developed using the PECO framework: Population—critically ill adult patients; Exposure—intestinal colonization by MDROs, ESBL‐E, CRE, or VRE; Comparator—no colonization; Outcomes—structural and compositional metrics of the intestinal microbiota. Full search strategies are provided in Appendix A. Supplementary studies were identified through backward and forward citation searching.

Records were screened independently by two reviewers (A.E.G. and D.C.L.) in two stages: screening of titles and abstracts and full‐text assessment against predefined eligibility criteria. Records with unclear eligibility were retained for full‐text review. Missing or unclear data prompted contact with the study authors. Discrepancies were resolved through discussion.

### 2.3 Data Extraction

Data were extracted sequentially by two reviewers (A.E.D. and D.C.L.). One reviewer was blinded to the study's objectives, while the other followed standardized extraction instructions to ensure consistency. From each included study, the following information was extracted: study metadata, methods, patients characteristics, ICU stay features, morbidity and mortality, colonization outcomes, and microbiota‐related data. Discrepancies were resolved through discussion between reviewers. If consensus could not be reached, a senior reviewer adjudicated (C.B.).

### 2.4 Synthesis and Statistical Analysis

We performed a multitiered synthesis integrating both quantitative and qualitative evidence to address the research question.

Quantitative data were combined using a random‐effects meta‐analysis, conducted by a statistician. When microbiota parameters were not numerically reported, data was extracted from digitized figures using WebPlotDigitizer version 4.8, with values closest to ICU admission extracted. If means and standard deviations were not reported, they were calculated from medians and interquartile ranges. Microbiota alterations in structure were reported using the α‐diversity complementary categories proposed by Cassol et al. (2025), and β‐diversity metrics, and those in composition were assessed by relative abundance of bacterial taxa. Subgroup analyses were conducted for patients colonized with VRE, ESBL‐E, or CRE. Statistical significance was defined as *p* < 0.05. Heterogeneity was quantified using the *I* ^2^ statistic (< 25% low, 25%–50% moderate, and > 50% high) (West 2010). Robustness was assessed via leave‐one‐out sensitivity analyses, systematically excluding individual studies. All quantitative analyses were performed using R environment for statistical computing and graphics (R Foundation for Statistical Computing, Vienna, Austria) version 4.3.2, with appropriate packages (meta, version 6.5.0) for meta‐analysis and data visualization (R: The R Project for Statistical Computing no date).

When quantitative synthesis was not possible, findings were reported narratively, focusing on recurring patterns and methodological rigor. Qualitative synthesis was performed by the research team with awareness of study objectives.

### 2.5 Data Quality and Publication Bias Assessment

Methodological quality of each study was evaluated using the Newcastle–Ottawa Scale by two independent reviewers (A.E.D. and D.C.L.) (Newcastle‐Ottawa Scale [NOS]|Ottawa Hospital Research Institute no date). Discrepancies were resolved through discussion. For critical appraisal and cross‐study comparability, the Strengthening the Organization and Reporting of Microbiome Studies (STORMS) checklist was applied to each study (Mirzayi et al. 2021). Publication bias was not assessed since the number of included studies was below 10.

## 3 Results

### 3.1 Study Selection

**Figure 1:**
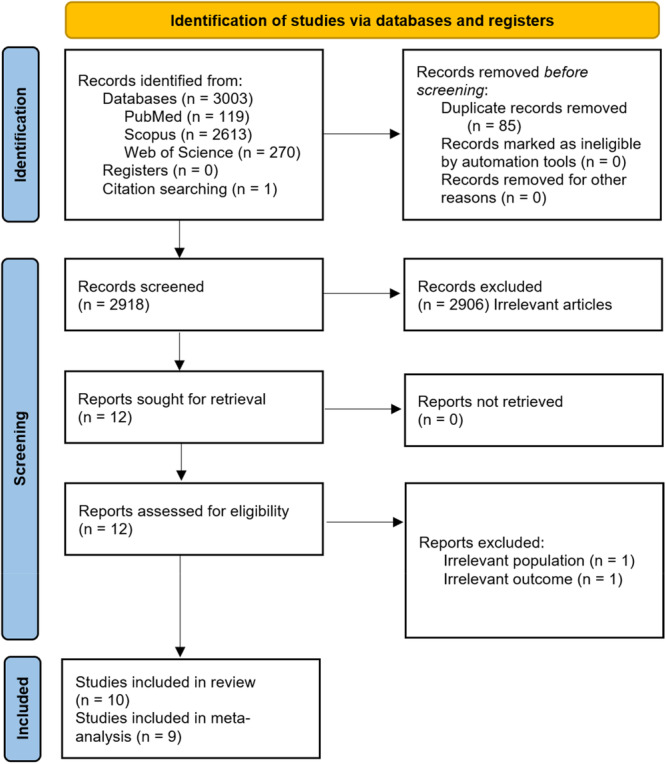
Preferred Reporting Items for Systematic Reviews and Meta‐Analyses flowchart diagram.

The literature search conducted on September 18, 2025 identified 3003 records from PubMed (*n* = 119), Web of Science (*n* = 270), Scopus (*n* = 2613), and one by citation searching. Ultimately, 11 studies including 2823 patients, published between 2019 and 2025, met the inclusion criteria. Two studies shared the same patient cohort, one of which was an in silico analysis (that was excluded); therefore, 10 studies contributed to the systematic review and nine to the meta‐analysis (Pettigrew et al. 2019; Collingwood et al. 2020; Fontaine 2020; Garcia et al. 2022; Sindi et al. 2022; Baek et al. 2023; García‐García et al. 2023; Prevel et al. 2023; Ling et al. 2025; Park et al. 2025). The study selection process is presented in the PRISMA flow diagram (Figure 1).

### 3.2 Study Characteristics

The 10 studies included in the systematic review originated from Europe, Asia, North America, and South America, and comprised nine prospective and one retrospective study. Colonized critically ill patients (*n* = 445) were compared with noncolonized ICU patients (*n* = 2225) in nine studies (Pettigrew et al. 2019; Collingwood et al. 2020; Fontaine 2020; Garcia et al. 2022; Sindi et al. 2022; Baek et al. 2023; García‐García et al. 2023; Prevel et al. 2023; Park et al. 2025), and with control patients (*n* = 153) in three papers (Pettigrew et al. 2019; Sindi et al. 2022; Ling et al. 2025). Study and patient characteristics are summarized in Appendix A, Table A1.

### 3.3 Patient Characteristics

Colonized patients were matched to comparator groups by age and/or gender in only three studies (Sindi et al. 2022; Prevel et al. 2023; Ling et al. 2025). Colonized patients were significantly older and/or had a higher burden of comorbidities in half of the studies (Pettigrew et al. 2019; Collingwood et al. 2020; Garcia et al. 2022; Prevel et al. 2023; Park et al. 2025). One study reported higher antibiotic exposure and more prior hospitalizations among colonized patients (Collingwood et al. 2020).

Patients were admitted to seven medical ICUs (Fontaine 2020; Garcia et al. 2022; Sindi et al. 2022; Baek et al. 2023; García‐García et al. 2023; Prevel et al. 2023; Park et al. 2025) and one mixed ICU (Pettigrew et al. 2019). The most common reasons for ICU admission were septic shock and respiratory failure (Baek et al. 2023; Prevel et al. 2023; Park et al. 2025). Severity scores were comparable between groups and indicated a moderate‐to‐high risk of in‐hospital mortality (Baek et al. 2023; García‐García et al. 2023; Prevel et al. 2023; Park et al. 2025). During ICU stay, exposure to various drugs was similar across groups. No consistent differences were observed in ICU‐related morbidity (Collingwood et al. 2020; Garcia et al. 2022; Sindi et al. 2022; Baek et al. 2023; García‐García et al. 2023; Park et al. 2025) or mortality (Garcia et al. 2022; Sindi et al. 2022; Baek et al. 2023; García‐García et al. 2023; Prevel et al. 2023) between colonized and comparator groups.

### 3.4 Systematic Review Findings

#### 3.4.1 Colonizing MDROs

Two studies focused on VRE (Collingwood et al. 2020; Park et al. 2025), three on CRE, including one on carbapenem‐resistant *Pseudomonas aeruginosa* (CRPA) (Pettigrew et al. 2019; Sindi et al. 2022; Baek et al. 2023), one on ESBL‐E (Prevel et al. 2023), and four evaluated patients colonized with a wide range of organisms, under the term of MDROs (Fontaine 2020; Garcia et al. 2022; García‐García et al. 2023; Ling et al. 2025). Only one study distinguished admission from ICU‐acquired colonization (Garcia et al. 2022).

Eight studies reported 402 colonizing bacterial isolates (Pettigrew et al. 2019; Collingwood et al. 2020; Fontaine 2020; Garcia et al. 2022; Baek et al. 2023; García‐García et al. 2023; Prevel et al. 2023; Park et al. 2025). VRE predominated (58.2%), followed by CRE (21.6%), ESBL‐E (13.4%), AmpC β‐lactamase–producing Enterobacterales (3.5%), and nonfermenting Gram‐negative bacteria (3.2%). CRE isolates were mainly *P. aeruginosa* and *Klebsiella pneumoniae* (Pettigrew et al. 2019; Baek et al. 2023), while for ESBL‐E, only *Escherichia coli* and *K. pneumoniae* were studied (Prevel et al. 2023).

#### 3.4.2 Microbiota Analysis

Microbiota profiling was performed on stool samples (Fontaine 2020; Sindi et al. 2022; Baek et al. 2023; Ling et al. 2025) or rectal swabs (Pettigrew et al. 2019; Collingwood et al. 2020; Garcia et al. 2022; García‐García et al. 2023; Prevel et al. 2023; Park et al. 2025). Time of sampling, as reported by seven of the studies, took place at ICU admission (Pettigrew et al. 2019; Collingwood et al. 2020; Fontaine 2020; Garcia et al. 2022; García‐García et al. 2023; Prevel et al. 2023; Park et al. 2025), within a 1‐week interval (Pettigrew et al. 2019; Collingwood et al. 2020; Fontaine 2020; Garcia et al. 2022; Park et al. 2025), and at ICU discharge (Pettigrew et al. 2019). The studies used polymerase chain reaction (PCR) amplification of 16S rRNA, with sequencing on Illumina platform, but targeted different hypervariable regions, V3 (Sindi et al. 2022), V3–V4 (Garcia et al. 2022; Sindi et al. 2022; Baek et al. 2023; Prevel et al. 2023; Ling et al. 2025; Park et al. 2025), V4 (Pettigrew et al. 2019; Collingwood et al. 2020; Fontaine 2020; Sindi et al. 2022), and V4–V5 (García‐García et al. 2023), and used different databases for taxonomic assignment.

α‐Diversity was assessed across all studies using metrics encompassing the four categories proposed by Cassol et al. (2025). Numerical values could be extracted for information (Shannon index), dominance/evenness (Simpson's index, and inverse Simpson [invSimpson]), and richness (Observed, and operational taxonomic unit [OTU]).

β‐Diversity was assessed in seven of the studies (Collingwood et al. 2020; Sindi et al. 2022; Baek et al. 2023; García‐García et al. 2023; Prevel et al. 2023; Ling et al. 2025; Park et al. 2025), with significant clustering between colonized and comparator groups reported in five studies (Collingwood et al. 2020; Sindi et al. 2022; Baek et al. 2023; Ling et al. 2025; Park et al. 2025). Numerical β‐diversity data could not be extracted for quantitative synthesis.

Microbiota composition was reported across multiple taxonomic levels. Despite heterogeneous analytical approaches, findings were consistent: colonized patients showed enrichment of opportunistic commensals, mainly Enterobacteriaceae family, most frequently reported genera being *Klebsiella* and *Enterococcus*, and depletion of SCFAs‐producing taxa (Appendix A, Tables A5, A6, A7, A8, A9).

For quantitative analysis, only numerical data at the phylum and family levels were suitable (Sindi et al. 2022; Baek et al. 2023; Ling et al. 2025).

#### 3.4.3 Additional Microbiota‐Related Findings

Several studies explored additional microbiota‐related outcomes, including associations with infection and mortality (Garcia et al. 2022; García‐García et al. 2023; Ling et al. 2025), metabolic and functional profiles (Baek et al. 2023; Ling et al. 2025), mycobiota composition (Prevel et al. 2023), SCFAs levels (Baek et al. 2023), and cytokine profiling (Ling et al. 2025).

Regarding infection, increased abundance of Enterobacteriaceae and Pseudomonadaceae was reported (Garcia et al. 2022; Ling et al. 2025), while Family XI was associated with lower odds of infection (Garcia et al. 2022). In terms of mortality, reduced Pielou's index, a metric of diversity, was associated with increased mortality risk (García‐García et al. 2023). Enterococcaceae and Pseudomonadaceae families were found enriched, whereas Prevotellaceae and Family XI were correspondingly depleted in patients with a fatal outcome (Garcia et al. 2022). In contrast, an abundance of *Corynebacterium* was associated with reduced mortality (García‐García et al. 2023).

Metabolic and functional profiling revealed consistent alterations in colonized patients in both studies reporting these data (Baek et al. 2023; Ling et al. 2025), notably increased activity of the phosphotransferase system, a pathway linked to multidrug resistance, and reduced sphingolipid metabolism, previously associated with inflammation and increased intestinal permeability [36, 37].

### 3.5 Meta‐Analysis Results

#### 3.5.1 MDROs‐Colonized Critically Ill Patients Compared With Noncolonized Patients

##### 3.5.1.1 Microbiota Structure (α‐Diversity)

###### Information: Shannon Diversity Index

**Figure 2:**
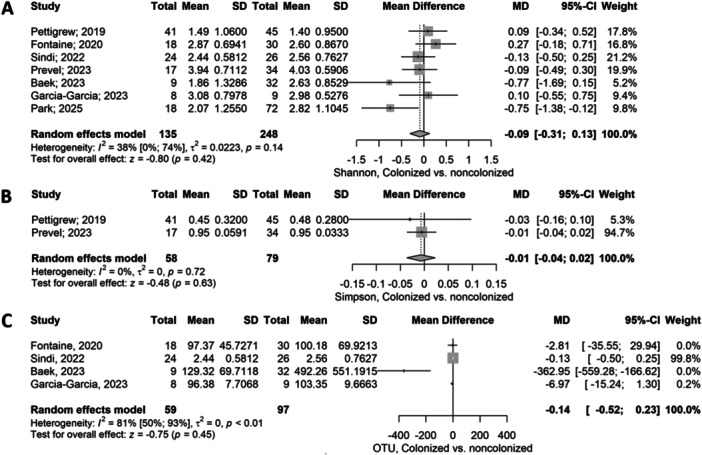
Microbiota structure in MDROs‐colonized versus noncolonized ICU patients. (A) Forest plot of Shannon index in MDROs‐colonized versus noncolonized ICU patients. (B) Forest plot of Simpson's index in MDROs‐colonized versus noncolonized ICU patients. (C) Forest plot of Observed species and OTU in MDROs‐colonized versus noncolonized ICU patients. CI, confidence interval; *I* ^2^, the percentage of variance attributable to study heterogeneity; ICU, intensive care unit; MD, mean difference; MDROs, multidrug‐resistant organisms; OTU, operational taxonomic unit; SD, standard deviation.

**Figure A1:**
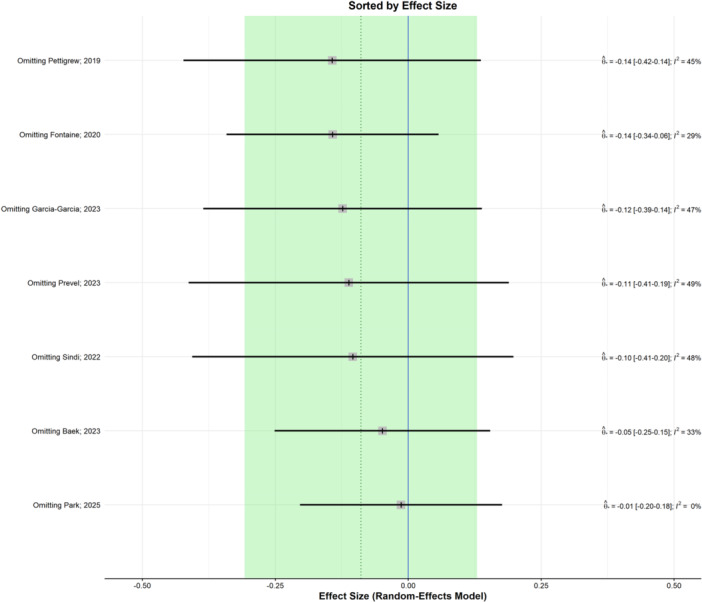
Leave‐one‐out sensitivity analysis plot for Shannon index in MDROs‐colonized versus noncolonized ICU patients; $\hat{\theta }$, meta‐analysis mean difference with 95% confidence interval; *I* ^2^, the percentage of variance attributable to study heterogeneity. ICU, intensive care unit; MDROs, multidrug‐resistant organisms.

The meta‐analysis of seven studies found colonized participants had nonsignificantly lower Shannon index values compared with the noncolonized (Figure 2). The heterogeneity, as measured by *I* ^2^, was moderate. A sensitivity analysis of leave‐one‐out type showed that no matter which study was excluded from meta‐analysis, the result remained nonsignificant (Appendix A, Figure A1). Excluding Park diminished heterogeneity to 0%, while excluding other studies kept the heterogeneity moderate.

###### Dominance/Evenness: Simpson's Diversity Index and invSimpson Index

The meta‐analysis of two studies found colonized participants had nonsignificantly lower Simpson's index values compared with the noncolonized (Figure 2). The heterogeneity was 0%. The invSimpson index had nonsignificantly lower values in the colonized group based on three studies (Table 1).

**Table 1:** Comparison of the invSimpson index and taxonomic abundance in MDROs‐colonized versus noncolonized ICU patients.

Characteristic	*N* studies	*N* MDROs‐colonized	*N* noncolonized	MD (95% CI)	*p* Value	*I* ^2^ (95% CI)	*p* Value	Studies
InvSimpson index	3	224	2031	−0.06 (95% CI −1.26 to 1.13)	0.323	75.7% (95% CI 19.8%–92.6%)	*p* = 0.016	Pettigrew et al. (2019), Collingwood et al. (2020), and García‐García et al. (2023)
Relative abundance, Pseudomonadota phylum	2	33	58	0.21 (0.04–0.39)	0.018	29.9 (NA–NA)	0.232	Sindi et al. (2022) and Baek et al. (2023)
Relative abundance, Bacteroidetes phylum	2	33	58	−0.2 (−0.63 to 0.23)	0.369	91.8 (71.5–97.6)	< 0.001	Sindi et al. (2022) and Baek et al. (2023)
Relative abundance, Bacillota phylum	2	33	58	−0.09 (−0.24 to 0.06)	0.247	0 (NA–NA)	0.799	Sindi et al. (2022) and Baek et al. (2023)
Relative abundance, Bifidobacteriaceae family	1	24	26	−0.11 (−0.19 to −0.02)	0.012	NC		Sindi et al. (2022)
Relative abundance, Coriobacteriaceae family	1	24	26	0 (−0.01 to 0)	0.046	NC		Sindi et al. (2022)
Relative abundance, Enterobacteriaceae family	1	24	26	0.37 (0.16–0.58)	< 0.001	NC		Sindi et al. (2022)

Abbreviations: CI, confidence interval; *I* ^2^, the percentage of variance attributable to study heterogeneity; ICU, intensive care unit; InvSimpson, inverse Simpson; MD, mean difference; MDROs, multidrug‐resistant organisms; *N*, number of participants; NC, cannot be computed due to the small number of studies.

###### Richness: Observed Species and OTU

**Figure A2:**
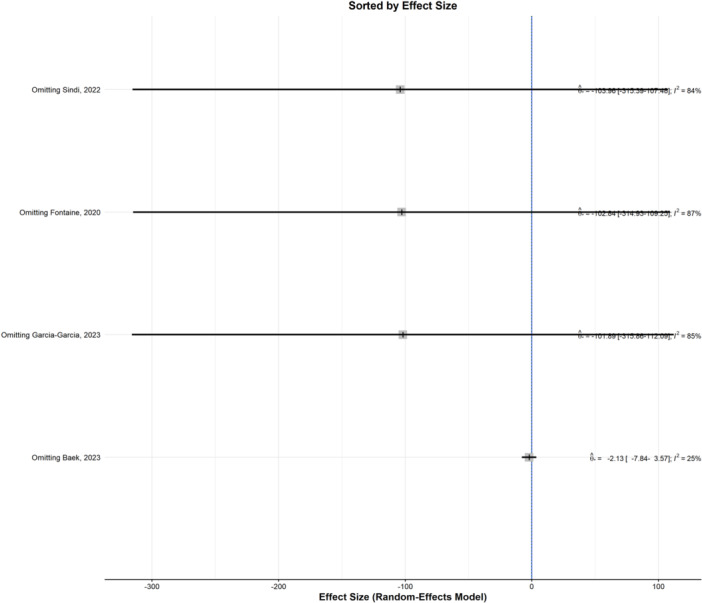
Leave‐one‐out sensitivity analysis for observed species/OTU in MDROs‐colonized versus noncolonized ICU patients; $\hat{\theta }$, meta‐analysis mean difference with 95% confidence interval; *I* ^2^, the percentage of variance attributable to study heterogeneity. ICU, intensive care unit; MDROs, multidrug‐resistant organisms; OTU, operational taxonomic unit.

The meta‐analysis of four studies found colonized participants had nonsignificantly lower Observed species/OTU values compared with the noncolonized (Figure 2). The heterogeneity was high. A sensitivity analysis of the leave‐one‐out type showed that no matter which study was excluded from meta‐analysis, the result remained nonsignificant (Appendix A, Figure A2). Excluding Baek reduced heterogeneity to moderate, whereas excluding other studies maintained high heterogeneity.

##### 3.5.1.2 Microbiota Composition (Relative Abundance of Bacterial Taxa)

Concerning the relative abundance of bacteria at the phylum level (Table 1), meta‐analyses of two studies found significantly higher relative abundance for the Pseudomonadota phylum for colonized. In case of relative abundance of the bacterial families, the Bifidobacteriaceae family had significantly lower values, and the Enterobacteriaceae family had significantly higher values in the colonized group.

#### 3.5.2 MDROs‐Colonized Critically Ill Patients Compared With Control Patients

##### 3.5.2.1 Microbiota Structure (α‐Diversity)

###### Information: Shannon Diversity Index

**Figure 3:**
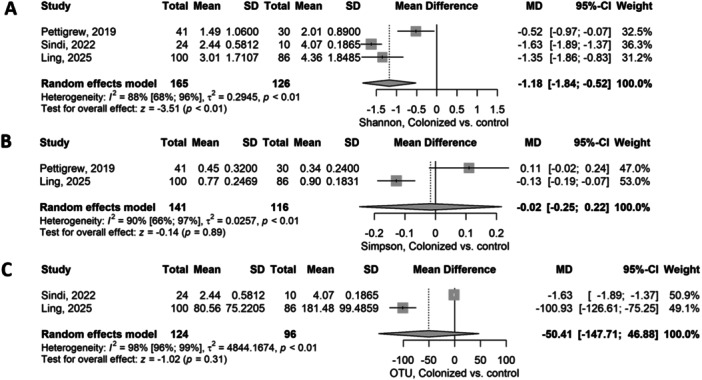
Microbiota structure in MDROs‐colonized ICU patients versus control patients. (A) Forest plot of Shannon index in MDROs‐colonized ICU patients versus control patients. (B) Forest plot of Simpson's index in MDROs‐colonized ICU patients versus control patients. (C) Forest plot of Observed species in MDROs‐colonized ICU patients versus control patients. CI, confidence interval; *I* ^2^, the percentage of variance attributable to study heterogeneity; ICU, intensive care unit; MD, mean difference; MDROs, multidrug‐resistant organisms; SD, standard deviation.

**Figure A3:**
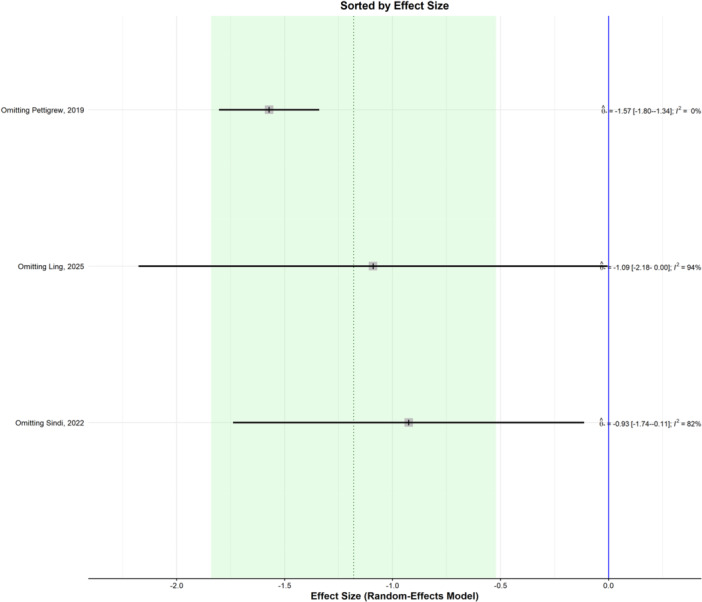
Leave‐one‐out sensitivity analysis plot for Shannon index in MDROs‐colonized ICU patients versus control patients; $\hat{\theta }$, meta‐analysis mean difference with 95% confidence interval; *I* ^2^, the percentage of variance attributable to study heterogeneity. ICU, intensive care unit; MDROs, multidrug‐resistant organisms.

The meta‐analysis of three studies found colonized patients had significantly lower Shannon index values compared with the control (Figure 3). Heterogeneity was high. Leave‐one‐out sensitivity analysis showed that removing whichever study, the results remain significant, with heterogeneity diminishing to 0 when omitting Pettigrew (Appendix A, Figure A3).

###### Dominance/Evenness: Simpson's Diversity Index and invSimpson Index

**Figure A4:**
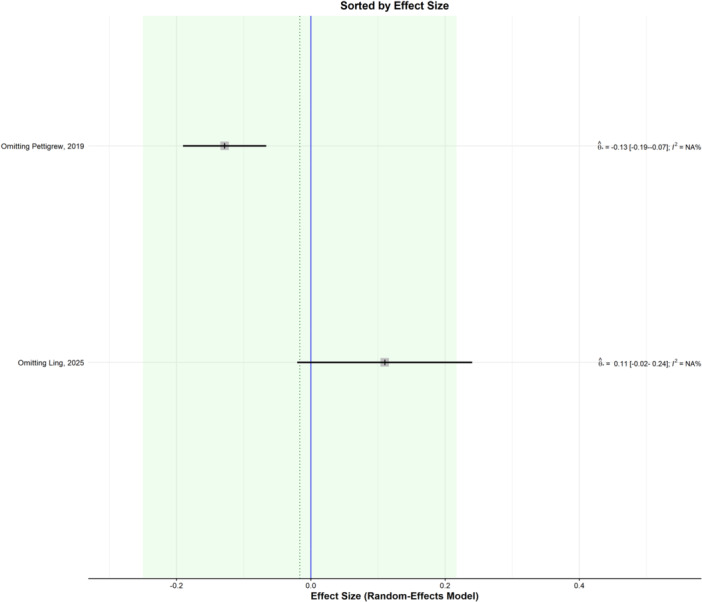
Leave‐one‐out sensitivity analysis plot for Simpson's index in MDROs‐colonized ICU patients versus control patients; $\hat{\theta }$, meta‐analysis mean difference with 95% confidence interval; *I* ^2^, the percentage of variance attributable to study heterogeneity. ICU, intensive care unit; MDROs, multidrug‐resistant organisms.

The meta‐analysis of two studies found colonized patients versus controls having conflicting Simpson index values, without being statistically significant (Figure 3). Assessment of heterogeneity found an *I* ^2^ high. Sensitivity analysis showed that omitting Pettigrew modified the results so that they were statistically significant, with lower values for Simpson's index in the colonized group (Appendix A, Figure A4). The invSimpson index had nonsignificantly lower values in the colonized group, and only one study provided data (Table 2).

**Table 2:** Comparison of the invSimpson index and taxonomic abundance in MDROs‐colonized ICU patients versus control patients.

Characteristic	*N* studies	*N* MDROs‐colonized	*N* control	MD (95% CI)	*p* Value	*I* ^2^ (95% CI)	*p* Value	Studies
invSimpson index	1	41	30	−0.98 (−3.52 to 1.56)	0.45	NC		Pettigrew et al. (2019)
Relative abundance, Pseudomonadota phylum	2	124	96	0.13 (0.02–0.25)	0.025	74.7 (0–94.3)	0.047	Sindi et al. (2022) and Ling et al. (2025)
Relative abundance, Bacteroidetes phylum	2	124	96	−0.07 (−0.21 to 0.08)	0.37	82.4 (26.2–95.8)	0.017	Sindi et al. (2022) and Ling et al. (2025)
Relative abundance, Bacillota phylum	2	124	96	0 (−0.04 to 0.03)	0.846	2.7 (NA–NA)	0.311	Sindi et al. (2022) and Ling et al. (2025)
Relative abundance, Bifidobacteriaceae family	2	124	96	−0.22 (−0.71 to 0.27)	0.373	83.9 (33.6–96.1)	0.013	Sindi et al. (2022) and Ling et al. (2025)
Relative abundance, Coriobacteriaceae family	2	124	96	−0.04 (−0.12 to 0.04)	0.331	69.1 (0–93)	0.072	Sindi et al. (2022) and Ling et al. (2025)
Relative abundance, Enterobacteriaceae family	2	124	96	0.34 (0.28–0.39)	< 0.001	0 (NA–NA)	0.373	Sindi et al. (2022) and Ling et al. (2025)

Abbreviations: CI, confidence interval; *I* ^2^, the percentage of variance attributable to study heterogeneity; ICU, intensive care unit; InvSimpson, inverse Simpon; MD, mean difference; MDROs, multidrug‐resistant organisms; MDROs, multidrug‐resistant organisms; *N*, number of participants; NC, cannot be computed due to the small number of studies.

###### Richness: Observed Species

**Figure A5:**
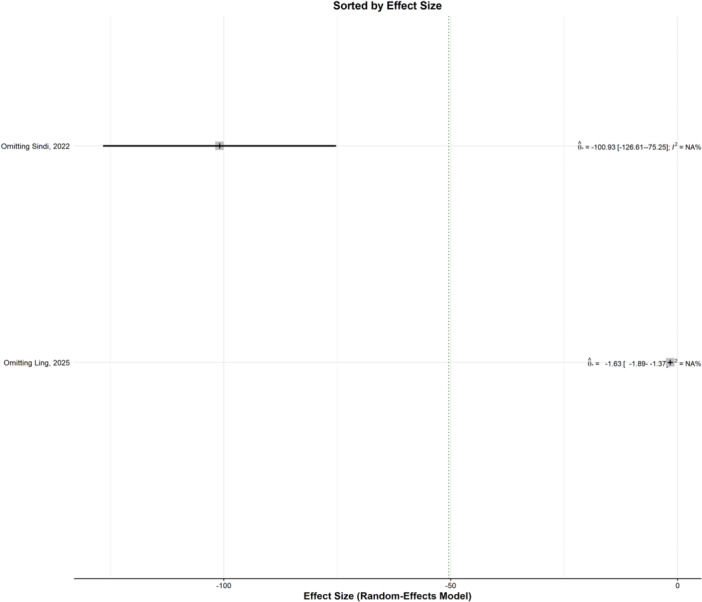
Leave‐one‐out sensitivity analysis plot for observed species in MDROs‐colonized ICU patients versus control patients; $\hat{\theta }$, meta‐analysis mean difference with 95% confidence interval; *I* ^2^, the percentage of variance attributable to study heterogeneity. ICU, intensive care unit; MDROs, multidrug‐resistant organisms.

The meta‐analysis of two studies revealed that colonized patients had nonsignificantly lower Observed species values compared with controls (Figure 3). The heterogeneity, as measured by *I* ^2^, was high. Leave‐one‐out sensitivity analysis showed that omitting either Sindi or Ling rendered the result significant, but in different directions (Appendix A, Figure A5).

##### 3.5.2.2 Microbiota Composition (Relative Abundance of Bacterial Taxa)

Concerning the relative abundance of bacterial phyla in colonized patients (Table 2), meta‐analyses of two studies found significantly higher relative abundance for the Pseudomonadota phylum, and for the Enterobacteriaceae family, the relative abundance was significantly higher.

#### 3.5.3 Subgroup Meta‐Analysis

##### 3.5.3.1 VRE‐Colonized Critically Ill Patients Compared With Noncolonized Patients

Two publications compared VRE‐colonized ICU patients with noncolonized, both in terms of microbiota alterations in structure, one study found significantly lower values for information (Shannon diversity index) and one found lower significant values for dominance/evenness (Simpson's index) in carriers (Appendix A, Table A2).

##### 3.5.3.2 CRE‐Colonized Critically Ill Patients Compared With Noncolonized and Control Patients

**Figure 4:**
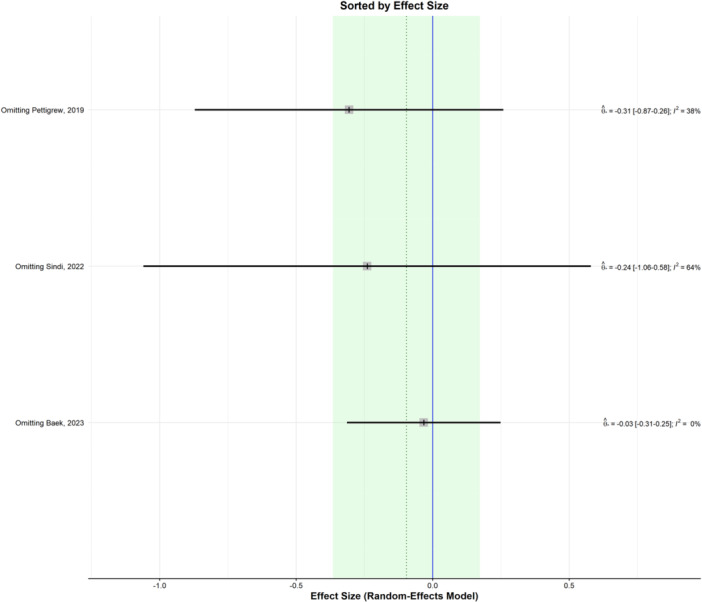
Leave‐one‐out sensitivity analysis plot for Shannon index in CRE‐colonized versus noncolonized ICU patients; $\hat{\theta }$, meta‐analysis mean difference with 95% confidence interval; *I* ^2^, the percentage of variance attributable to study heterogeneity. CRE, carbapenem‐resistant Enterobacterales; ICU, intensive care unit.

Comparison of Shannon index in colonized patients with CRE versus noncolonized in a meta‐analysis of three studies, found nonsignificantly lower values in carriers, the result maintained even after omitting whichever study in the sensitivity analysis (Figure 4, Appendix A, Table A3). The relative abundances at the phylum and family level were reported, with significantly higher values for Pseudomonadota phylum, significantly lower values for Bifidobacteriaceae family and Coriobacteriaceae family, and higher and significant values for Enterobacteriaceae family (Appendix A, Table A3).

When compared with control group, the meta‐analysis of two studies found Shannon index value significantly lower in colonized. OTU value was found significantly lower in carriers as well (Appendix A, Table A4). The relative abundances at the phylum and family level were reported in one study, with significantly higher values for the Pseudomonadota phylum, significantly lower values for Bifidobacteriaceae and Coriobacteriaceae families, and higher and significant values for the Enterobacteriaceae family (Appendix A, Table A4).

##### 3.5.3.3 ESBL‐E‐Colonized Critically Ill Patients Compared With Noncolonized Patients

In one study, colonized patients with ESBL‐E were specifically compared with matched noncolonized patients, and data on microbiota structure were reported. Both information (Shannon index) and dominance/evenness (Simpson's index) were lower in the *K. pneumoniae*‐colonized group, whereas in the *E. coli*‐colonized group, these measures were slightly higher compared with noncolonized patients (Table 3).

**Table 3:** Comparison of the Shannon index and Simpson's index between ESBL‐E‐colonized and noncolonized ICU patients.

Characteristic	*N* studies	*N* ESBL	*N* noncolonized	Effect size (95% CI)	*p* Value	*I* ^2^ (95% CI)	*p* Value	Studies
Shannon, *E. coli* colonized versus noncolonized	1	11	22	0.17 (−0.18 to 0.52)	0.348	NC		Prevel et al. (2023)
Simpson, *E. coli* colonized versus noncolonized	1	11	22	0.02 (0–0.04)	0.033	NC		Prevel et al. (2023)
Shannon, *K. pneumoniae*‐colonized versus noncolonized	1	6	12	−0.58 (−1.39 to 0.24)	0.164	NC		Prevel et al. (2023)
Simpson, *K. pneumoniae*‐colonized versus noncolonized	1	6	12	−0.05 (−0.12 to 0.02)	0.182	NC		Prevel et al. (2023)

Abbreviations: CI, confidence interval; *E. coli*, *Escherichia coli*; ESBL‐E, extended‐spectrum β‐lactamase–producing Enterobacterales; *I* ^2^, the percentage of variance attributable to study heterogeneity; ICU, intensive care unit; *K. pneumoniae*, *Klebsiella pneumoniae*; MD, mean difference; *N*, number of participants; NC, cannot be computed due to the small number of studies.

### 3.6 Risk of Bias and Study Quality

The methodological quality of the included studies was assessed with the NOS (Table 4). Case and control definitions were adequate in all the studies. Four (44.4%) of the studies had a representativeness of cases, using consecutive sampling; all the others (55.6%) were unclear in specifying participant selection. Almost all studies (88.9%) had an adequate selection of controls. Three studies had improved comparability by using matching for age and gender; the other six (66.7%) did not use any method in this respect. Exposure was correctly measured with adequate laboratory methods and techniques.

**Table 4:** Methodological quality of selected studies assessed with the Newcastle‐Ottawa scale (NOS).

Kind of study	No study	Selection CD|RC|SC|DC	Comparability	Exposure	Prior hospitalization^a^	Prior antibiotic therapy
Case–control	Prével et al. (2023)	*|*|*|*	*(age, gender)	*	*	*
Case–control	Collingwood et al. (2020)	*|*|*|*	—	*	*	—
Cross‐sectional	Pettigrew et al. (2019)	*|*| */*|*	—	*	—	—
Cross‐sectional	Park et al. (2025)	*|–|*|*	—	*	—	—
Cross‐sectional	Baek et al. (2023)	*|–|*|*	—	*	—	*
Cross‐sectional	García‐García et al. (2023)	*|–|*|*	—	*	*	—
Cross‐sectional	Ling et al. (2025)	*|–|–|*	*(age, gender)	*	—	—
Cross‐sectional	Sindi et al. (2022)	*|–|–/*|*	*(age)	*	—	—
Cross‐sectional	Fontaine et al. (2020)	*|*|*|*	—	*	—	*
	Rubio Garcia et al. (2022)		—	*	*	—

*Note:* For the confounding domain, the confounders are listed; in case there were multiple groups that were compared, multiple assessments are separated by |. *indicate a criterion that this study meets adequately.

Abbreviations: CD, case definition adequate; DC, definition of controls; RC, representativeness of the cases; SC, selection of controls.

^a^ Additional bias indicators, besides those in NOS.

Study quality was evaluated using the STORMS checklist to examine reporting transparency and enable cross‐study comparability (Mirzayi et al. 2021). STORMS checklist coverage ranged from 42% in Sindi et al. to 71% in Ling et al. across the 10 studies. None of the seven studies published after 2021 formally adopted STORMS. Persistent deficiencies were observed in reproducibility, transparency of sampling and exposure, quality assurance, and robustness checks, with methodological caveats not consistently addressed (Appendix A, Table A10).

## 4 Discussion

The independent association between intestinal microbiota alterations and MDROs‐colonization in ICU patients was not assessed by any of the included studies.

Age and/or gender were infrequently controlled for, with only three studies performing matching (Sindi et al. 2022; Prevel et al. 2023; Ling et al. 2025). Colonized ICU patients were older, with mean age differences of approximately 10 years, in two studies (Garcia et al. 2022; Park et al. 2025). Population‐based data suggest that substantial age‐related microbiota changes are more evident in the young and in those older than 70 years of age (Biagi et al. 2011; de la Cuesta‐Zuluaga et al. 2019), indicating, although statistically significant, a difference of 10 years may not reflect a biologically meaningful effect. Groups were not matched for comorbidities, closely associated with dysbiosis (Wilkins et al. 2019; Shen et al. 2025). Patients colonized by VRE, CRE, and ESBL‐E, had greater comorbidity burden, greater antibiotic exposure or longer history of prior hospitalization, but findings regarding α‐ and β‐diversity across indices and comparator groups were inconsistent (Pettigrew et al. 2019; Collingwood et al. 2020; Garcia et al. 2022; Baek et al. 2023; Prevel et al. 2023), with only two studies reporting significant structural microbiota alterations in more comorbid populations (Pettigrew et al. 2019; Baek et al. 2023). Race or body mass index are linked to microbiota as well, but there were no differences across studies (Mallott et al. 2023; Li et al. 2024). These differences limit though the attribution of microbiota changes to colonization itself.

Intestinal microbiota assessment varied in sample type, timing, and analytical approach. While rectal swabs are a practical alternative in the ICU setting, microbial compositional differences were demonstrated between unsoiled rectal swabs and stool, including differential representation of anaerobes (Bansal 2018; Fair et al. 2019). None of the included papers that used swabs reported their characteristics, and only one of the papers validated the rectal swabs as a surrogate for stool samples on a subset of patients (Garcia et al. 2022). A pragmatic consensus needs to be reached before the exclusion of any of the samples can be applied in further studies. Sampling timing also differed substantially, with no baseline data collected prior to ICU stay, specimens being obtained at admission, during the ICU stay, or at discharge. Most studies combined at‐admission and acquired colonization, limiting discrimination between microbiota features associated with early MDROs‐carriage and changes occurring during ICU exposure and precluding assessment of time‐dependent microbiota alterations according to colonization status. Although all studies used PCR amplification of 16S rRNA gene and sequencing on Illumina platform, we acknowledge that heterogeneity in targeted sequencing regions, and bioinformatic pipelines may limit direct comparability across studies and should be considered when interpreting pooled findings (Bose and Moore 2023; Xu et al. 2023). However, the primary aim of our review was not to provide an exhaustive characterization of the microbiota in critically ill patients, but rather to identify recurring differences between colonized and noncolonized/control patients. Patterns consistent with what is known about the microbiota in ICU patients were reported, including similar diversity trends and recurrent compositional shifts, reflecting the reliability of the findings rather than the different methodologies used.

Concerning microbiota structure, compared with ICU noncolonized patients, MDROs‐colonized patients had generally lower information (Shannon index), dominance/evenness (Simpson's index, invSimpson), and richness (observed species/OTUs), with no significant differences reported in studies (Pettigrew et al. 2019; Collingwood et al. 2020; Fontaine 2020; Sindi et al. 2022; Baek et al. 2023; García‐García et al. 2023; Prevel et al. 2023; Ling et al. 2025; Park et al. 2025). When compared with control groups, ICU MDROs‐colonized patients demonstrated altered microbiota structure, having significantly lower information. Reported as significantly reduced in studies involving CRPA, CRE, or mixed MDROs‐colonization (Pettigrew et al. 2019; Sindi et al. 2022; Ling et al. 2025), a lower degree of entropy was previously stated in ICU patients. Critically ill patients were found to present communities consisting of just 2–4 multidrug‐resistant pathogens (Zaborin et al. 2014), with loss of protective commensals anaerobes and overrepresentation of potential pathogens (Enterococcaceae family and Enterobacterales order) (Vincent et al. 2013; Livanos 2018; Rao 2020). Loss of local specificity and abundance of the same taxon in multiple sites, with subsequent infection, were also reported (McDonald 2016; Ojima et al. 2016; Yeh et al. 2016; Ravi et al. 2019). Dominance/evenness and richness were as well lower in carriers, but insignificant when compared with a control group. These pooled results have to be interpreted with caution. Not all three studies that compared carriers with controls recruited healthy subjects for the control group; Pettigrew et al. (2019) recruited noncolonized ICU patients without antibiotic exposure, but sensitivity analyses confirmed the robustness of the findings, with significantly lower information in carriers, regardless of which study was omitted.

Microbiota composition was assessed in three studies (Sindi et al. 2022; Baek et al. 2023; Ling et al. 2025). Compared with noncolonized patients, MDROs‐colonized ICU patients showed significantly higher relative abundance of Pseudomonadota phylum and Enterobacteriaceae family and significantly lower abundance of Bifidobacteriaceae family (Sindi et al. 2022; Baek et al. 2023). Similar patterns were observed when compared with control groups (Sindi et al. 2022; Ling et al. 2025). Enterobacteriaceae family expansion is a common pattern in an altered microbiota composition, being also reported in non‐ICU hospitalized MDROs‐carriers (Araos et al. 2017; Korach‐Rechtman 2020), and associated with intestinal and extra‐intestinal diseases and seen as a potential marker to detect the onset and/or progression of disease (Moreira De Gouveia et al. 2024). Reduced abundance of Bifidobacteriaceae family reflects loss of commensal taxa accompanying pathogen expansion and was similarly observed in hospitalized MDROs‐carriers and related to other disease progression (Hidalgo‐Cantabrana 2017), whereas higher abundances of Bifidobacteriaceae and other commensals, including Lactobacillaceae, were described in patients who remained uncolonized (Araos et al. 2016; Annavajhala et al. 2019).

An additional consideration is the relevance of resistance phenotype and bacterial species when evaluating microbiota in MDROs‐colonized patients. In this review, alterations in both microbiota structure and composition were observed in patients colonized with VRE (Collingwood et al. 2020; Park et al. 2025). In CRE‐carriers, differences were limited to compositional changes, the subgroup including multiple bacterial species. Pettigrew et al. reported no clear microbiota alterations among CRPA‐carriers, apart from an isolated difference in Shannon diversity when compared with controls (Pettigrew et al. 2019). This finding may reflect study design limitations, as samples from noncolonized and controls were temporally matched to precolonization rather than confirmed colonization samples. For ESBL‐colonized patients, our extracted data indicated a modest difference in Simpson's diversity between *E. coli* colonized and noncolonized patients, which was not reported in the original study (Prevel et al. 2023). This discrepancy may be attributable to the estimation of values from published figures and highlights the need for further studies with a larger sample size to confirm these findings. Prével et al. underscored however species‐specific effects, reporting lower microbiota diversity in ESBL‐*K. pneumoniae* carriers compared with ESBL‐*E. coli* carriers (Prevel et al. 2023). Comparable observations have been reported in nursing home populations, where no differences in microbiota structure were observed when MDROs‐carriage was broadly defined, whereas analyses restricted to ESBL‐E revealed reduced richness and distinct β‐diversity in carriers (Le Bastard et al. 2020; Ducarmon et al. 2021).

Several limitations of this review should be acknowledged. All included studies were observational, precluding causal inference; the temporal direction of the searched association remained uncertain. Many studies were single‐center with small sample sizes, increasing susceptibility to center‐specific practices. Residual confounding is likely. Matching for major determinants of microbiota was inconsistent: only three studies matched for age and/or gender. Substantial heterogeneity also existed in the definition and analysis of MDROs‐colonization. Studies variably grouped multiple bacterial species and resistance phenotypes or focused on specific organism–resistance combinations. Although stratified analyses suggested phenotype‐ and species‐specific microbiota patterns, the limited number of studies precluded robust subgroup meta‐analyses and may have diluted organism‐specific signals. Methodological variability further limited comparability. Differences in sample type, timing of sampling relative to ICU admission, variability in targeted regions, bioinformatic pipelines, and diversity metrics, frequent pooling of baseline and acquired colonization, constrained quantitative synthesis, restricted interpretation of microbiota changes and may have underestimated microbiota alterations. Finally, numerical values extracted from figures introduced potential measurement error, and statistical heterogeneity was high. Although sensitivity analyses supported the robustness of some findings, underlying sources of heterogeneity could not be fully resolved.

Among the strengths of the study is that, to our knowledge, it is the first systematic review and meta‐analysis to examine the association between intestinal microbiota alterations and MDROs‐colonization specifically in adult critically ill patients, addressing an important gap in the critical care literature. Clinical relevance is enhanced by the exclusive focus on critically ill patients, a population at high risk for MDROs‐colonization and infection with substantial implications for healthcare systems. Despite methodological and clinical heterogeneity, consistent patterns emerged across studies, with reduced microbiota diversity and enrichment of Pseudomonadota phylum and Enterobacteriaceae family in MDROs‐colonized patients. At present, microbiota‐based markers could guide targeted surveillance for MDROs, supporting early identification of high‐risk patients and informing infection prevention strategies. Where data permitted, stratified analyses by resistance phenotype or bacterial species revealed more pronounced microbiota differences, underscoring the importance of organism‐specific approaches and providing a framework for defining reproducible microbiota endpoints in future critical care research. Stratification by resistance phenotype or bacterial species may further refine risk assessment, allowing more personalized infection control measures. Reporting followed recent recommendations for microbiota‐based research. Microbiota structure was analyzed using complementary diversity dimensions (richness, dominance/evenness, and information), allowing a structured and transparent characterization of alterations. In addition, the STORMS checklist was applied to all studies, facilitating standardized assessment of microbiome‐related reporting and highlighting key areas for improvement.

## 5 Conclusions

MDROs‐colonization in critically ill patients is consistently associated with altered intestinal microbiota structure and composition, notably reduced entropy (information), with expansion of Enterobacteriaceae family. α‐Diversity metrics like the Shannon index may inform targeted surveillance of MDROs‐colonization status and infection‐prevention strategies, although causality remains to be established. Future research should focus on longitudinal, multicenter studies, using standardized approaches, to clarify temporal dynamics and define causal relationships between specific MDROs‐colonization and intestinal microbiota alterations, ultimately aiming to modulate intestinal microbial communities to reduce colonization and subsequent infection in the most vulnerable of patients.

## Author Contributions

**Andra‐Elena Goicea:** conceptualization, investigation, writing – original draft, methodology, visualization, writing – review and editing, project administration, data curation. **Daniel‐Corneliu Leucuța:** conceptualization, investigation, writing – original draft, methodology, visualization, writing – review and editing, formal analysis, software, data curation. **Constantin Bodolea:** conceptualization, supervision, validation, writing – review and editing.

## Funding

The authors have nothing to report.

## Ethics Statement

The authors have nothing to report.

## Conflicts of Interest

None declared.

## Data Availability

All data generated or analyzed during this study are included in this published article.

## Appendix A

**Search Strategy**

**PubMed**

((microbiota[MeSH Terms]) OR “Microbial community” [All Fields] OR (microbiome [MeSH Terms]) OR “microbiome” [All Fields] OR “bacteria” [MeSH Terms] OR “bacteria” [All Fields])

AND (“colonization” [All Fields] OR “colonizing” [All Fields]) AND ((care, critical [MeSH Terms]) OR “critical care” OR “intensive care” (care, intensive [MeSH Terms]) OR (critically ill [MeSH Terms]) OR “critically ill” OR “Intensive Care Units” [MeSH Terms] OR “Intensive Care Units” [All Fields]) AND (“Drug Resistance, Microbial” [MeSH Terms] OR ((“Drug” [All Fields] OR “Antibiotic” [All Fields] OR “Antimicrobial” [All Fields]) AND (“resistance” [All Fields])) OR MDRO [All Fields] OR ESBL [All Fields] OR MRSA [All Fields] OR (“resistance” [All Fields])) AND (“RNA, Ribosomal, 16S” [MeSH Terms] OR (“RNA” [All Fields] AND “ribosomal” [All Fields]) OR “16S rRNA” [All Fields] OR “DNA, Bacterial” [MeSH Terms] OR (“DNA” [All Fields] AND (“bacteria” [All Fields] OR “bacterial” [All Fields])) OR “Sequence Analysis” [MeSH Terms] OR “Sequence Analysis” [All Fields] OR “sequencing” [All Fields] OR “genome” [MeSH Terms] OR “genome” [All Fields] OR “metagenome” [MeSH Terms] OR “metagenome” [All Fields]) NOT (neonatal [All Fields] OR children [All Fields] OR NICU [All Fields] OR neonates [All Fields])

**Web of Science**

TS = (((microbiota OR “microbial community” OR microbiome OR bacteria)) AND (“critical care” OR “intensive care” OR “critically ill” OR ICU OR “intensive care unit*” OR ITU OR “high dependency unit*” OR HDU) AND (((antibiot* OR antimicrob* OR “anti‐microb*” OR drug*) NEAR/3 resist*) OR AMR OR “antimicrobial resist*” OR “antibacterial resist*” OR “multidrug resist*” OR “multi‐drug resist*” OR MDR OR MDRO OR ESBL OR “extended‐spectrum beta‐lactamase*” OR MRSA OR VRE OR CRE OR “carbapenem‐resistan*” OR KPC OR NDM OR OXA‐48*) AND (“16S rRNA” OR (“ribosomal” NEAR/3 RNA) OR (sequenc* NEAR/3 (16S OR metagenom* OR shotgun OR amplicon*)) OR metagenom* OR genom* OR “whole genome” OR WGS OR “next generation sequenc*” OR “high‐throughput sequenc*”) NOT (neonat* OR child* OR infant* OR pediatric* OR pediatric* OR NICU OR PICU)) ALL(((microbiota OR “microbial community” OR microbiome OR bacteria)) AND (“critical care” OR “intensive care” OR “critically ill” OR ICU OR “intensive care unit*” OR ITU OR “high dependency unit*” OR HDU) AND (((antibiot* OR antimicrob* OR “anti‐microb*” OR drug*) NEAR/3 resist*) OR AMR OR “antimicrobial resist*” OR “antibacterial resist*” OR “multidrug resist*” OR “multi‐drug resist*” OR MDR OR MDRO OR ESBL OR “extended‐spectrum beta‐lactamase*” OR MRSA OR VRE OR CRE OR “carbapenem‐resistan*” OR KPC OR NDM OR OXA‐48*) AND (“16S rRNA” OR (“ribosomal” NEAR/3 RNA) OR (sequenc* NEAR/3 (16S OR metagenom* OR shotgun OR amplicon*)) OR metagenom* OR genom* OR “whole genome” OR WGS OR “next generation sequenc*” OR “high‐throughput sequenc*”) NOT (neonat* OR child* OR infant* OR pediatric* OR pediatric* OR NICU OR PICU))

**Scopus**

ALL (((microbiota) OR “Microbial community” OR “microbiome” OR “bacteria”) AND (“colonization” OR “colonizing”) AND (“critical care” OR “intensive care” OR “critically ill” OR “Intensive Care Units”) AND (((“Drug” OR “Antibiotic” OR “Antimicrobial”) AND “resistance”) OR MDRO OR ESBL OR MRSA OR “resistance”) AND ((“RNA” AND “ribosomal”) OR “16S rRNA” OR (“DNA” AND (“bacteria” OR “bacterial”)) OR “Sequence Analysis” OR “sequencing” OR “genome” OR “metagenome”) AND NOT (neonatal OR children OR NICU OR neonates)) AND (LIMIT‐TO (DOCTYPE, “ar”)) AND (LIMIT‐TO (EXACTKEYWORD, “Human”)) AND (LIMIT‐TO (SRCTYPE, “j”)) AND (LIMIT‐TO (SUBJAREA, “MEDI”))

**Table A1:** Study and patients' characteristics.

Study name	Prével	Collingwood	Pettigrew	Park	Baek
Year	2023	2020	2019	2025	2023
Region, country	Europe, France	North America, USA	North America, USA	South America, Columbia	South Asia, Korea
Study design	Prospective	Prospective	Prospective	Prospective	retrospective
Population enrollment period	October 2019–March 2020	July 2014–October 2014, January 2016–May 2016	September 2001–March 2009	October 2019–November 2023	October 2016–August 2021
Inclusion and exclusion criteria	I: > 18 yo ICU patients	I: Adult ICU patients	I: Adult ICU patients	I: Adult ICU patients with sepsis receiving broad‐spectrum antibiotics within 24 h of ICU admission; E: COVID19+, do not resuscitate/no escalation of care orders	I: > 18 yo ICU patients with septic shock/respiratory failure
Comparison case/control	ESBL‐E‐carriers (17)/matched noncarriers (34)	VRE‐carriers (175)/noncarriers (1977)	CRPA‐carriers (41)/noncarriers (45)/control (23)	VRE‐carriers (18)/noncarriers (72)	CRE‐carriers (9)/noncarriers (32)
Control definition	Noncarriers ICU patients matched on gender and age ±5 years	Noncarriers ICU patients	(1) Noncarriers ICU patients; (2) ICU patients that did not receive antibiotics and did not get colonized during ICU stay	Noncarriers ICU patients	Noncarriers ICU patients
Age (years), mean (SD)/median (IQR) [range] case/control	ESBL‐*E. coli*‐carriers/noncarriers: 77 (66–84)/74 (65–78); ESBL‐*Klebsiella pneumoniae*‐carriers/noncarriers: 76 (57–84)/74 (66–81)	2014: 56.34 (+15.03)/57.36 (+16.81); 2016: 58.90 (+16.08)/58.69 (+16.38)	Carriers/noncarriers, 52.8 (14.8)/54.8 (11.9); carriers/control, 52.8 (14.8)/52 (14.7)	70 (62–76)/60 (44–70)	64 (55–69)/64.5 (54–69)
Female (%) case/control	ESBL‐*E. coli*‐carriers/noncarriers: 29.41%/29.41%; ESBL *K. pneumoniae*‐carriers/noncarriers: 16.7%/16.7%	2014: 42.5%/47.9%; 2016: 40.2%/44.5%	Carriers/noncarriers: 41.5%/40%; carriers/control: 41.5%/47.8%	28%/49%	33.33%/28.1%
Race (%) case/control		2014: White 79.5%/82.3%, Black 17.8%/9.4%, Other 2.7%/8.2%; 2016: White 83.3%/86%, Black 11.8%/9%, Other 4.9%/5%	Carriers/noncarriers: White 37.5%/51.1%, Black 55%/46.7%, Other 7.5%/2.2%; carriers/control: White 37.5%/54.6%, Black 55%/45.4%, Other 7.5%/0%	White 50%/36%, Black 17%/22%, Other 33.33%/41.7%	
BMI (kg/m^2^), mean (SD)/median (IQR) [range] case/control					23 (21–26.3)/22.7 (18.6–24.4)
Comorbidity index (score), mean (SD)/median (IQR) [range] case/control		Elixhauser: 2014 13.39 (+9.28)/6.52 (+8.57); 2016 11.34 (+9.41)/6.43 (+7.22)	Elixhauser: carriers/noncarriers 1 (2)/1 (2); carriers/control 1 (2)/2 (2); Charlson: carriers/noncarriers 4 (3)/4 (2); carriers/control 4 (3)/4 (3)	Charlson: 5 (4–5)/3 (2–6)	
Hospitalization history (%) case/control	ESBL‐*E. coli*‐carriers/noncarriers: 54.54%/59.09%; ESBL‐*K. pneumoniae*‐carriers/noncarriers: 83.33%/58.33%	2014: 54.8%/57.9%; 2016: 44.1%/54.9%			88.9%/50%
Antibiotic therapy (%) case/control	ESBL‐*E. coli*‐carriers/noncarriers: 36.26%/40.9%; ESBL‐*K. pneumoniae*‐carriers/noncarriers: 33.33%/25%	2014: 13.7%/9.4%; 2016: 35.2%/11.8%			55.6%/28.1%
Type of ICU	Medical	Not specified	Mixed	Medical	Medical
ICU Admission Severity Scoring Systems (score), mean (SD)/median (IQR) [range] case/control	SAPS II: ESBL‐*E. coli*‐carriers/noncarriers 55 (31–68)/70 (45–78); ESBL‐*K. pneumoniae*‐carriers/noncarriers 80 (68–87)/65 (54–77)			SOFA: 9 (7–10)/8 (6.5–10)	SOFA: 13 (7.5–15)/12 (7–15.5), APACHE II: 31 (22–39)/25 (19–32.5)
Cause of ICU admission (%) case/control	septic shock: 29.41%/38.23%; ARDS: 11.8%/17.64%			Sepsis 100%/100%	Septic shock 55.6%/43.8%, respiratory failure 44.4%/56.3%
H2 blocker or proton pump inhibitor (PPI) (%) case/control	PPI: 17.64%/32.35%		PPI: carriers/noncarriers 80.5%/82.2%; carriers/control 80.5%/91.3%		100%/90.6%
Opioids (%) case/control			Carriers/noncarriers: 75.6%/75.6%; carriers/control: 75.6%/65.2%		55.6%/62.5%
Steroids (%) case			Carriers/noncarriers: 39%/37.8%; carriers/control: 39%/21.7%		
Vasoactive agent (%) case/control				72%/72%	77.8%/53.1%
Antibiotic (%) case/control			Carriers/noncarriers: 82.9%/82.2%; carriers/control: 82.9%/0%	100%/100%	
β‐Lactam class (%) case/control			Carriers/noncarriers: 75.6%/64.4%; carriers/control: 75.6%/0%		100%/96.9%
Quinolone class (%) case/control			Carriers/noncarriers: 26.8%/42.2%; carriers/control: 26.8%/0%		33.3%/46.9%
Piperacillin‐tazobactam class (%) case/control			Carriers/noncarriers: 56.1%/26.7%; carriers/control: 56.1%/0%		22.2%/46.9%
Macrolide class (%) case/control			Carriers/noncarriers: 12.2%/8.9%; carriers/control: 12.2%/0%		
Vancomycin class (%) case/control			Carriers/noncarriers: 48.8%/40%; carriers/control: 48.8%/0%		
Carbapenem class (%) case/control					66.7%/50%
Artificial nutrition (%) case/control					88.9%/62.5%
Infection (%) case/control		2014: *K. pneumoniae* clinical culture 1.4%/2.5%, *K. pneumoniae* infection 1.4%/1.2%; 2016: *K. pneumoniae* clinical culture 1%/1.7%, *K. pneumoniae* infection 1%/0.8%			Bacteremia 55.6%/40.6%
Mechanical ventilation (%) case/control				67%/83%	77.8%/93.8%
Renal replacement therapy (%) case/control					55.6%/53.1%
Length of ICU stay (days), mean (SD)/median (IQR) [range] case/control					17 (8.5–38)/14.5 (8–45)
Length of hospital stay (days), mean (SD)/median (IQR) [range] case/control					60 (33.5–91.5)/50 (22–129)
ICU‐mortality (%) case/control	17.64%/23.52%				22.2%/21.9%
In‐hospital mortality (%) case/control					44.4%/34.4%
AmpC β‐lactamase–producing‐E					
ESBL‐E	17				2
CRE			41		9
VRE		175		18	2
Nonfermenter GNB					
*AmpC* β‐lactamase‐*Hafnia alvei*					
AmpC β‐lactamase‐*Morganella morganii*					
AmpC β‐lactamase‐*Enterobacter aerogenes*					
AmpC β‐lactamase‐*Enterobacter cloacae*					
AmpC β‐lactamase‐*Citrobacter freundii*					
AmpC β‐lactamase‐*Citrobacter farmeri*					
ESBL‐*K. pneumoniae*	6				
ESBL‐*E. coli*	11				
ESBL‐*C. freundii*					
CR‐*K. pneumoniae*					7
CR‐*E. coli*					1
CR‐*K. oxytoca*					1
CR‐*E. cloacae*					
CR‐*Serratia marcescens*					
CR‐*Pseudomonas aeruginosa*			41		
NFGNB‐*Stenotrophomonas maltophilia*					
NFGNB‐*P. aeruginosa*					
Ambler Class A (KPC enzyme)					4
Ambler Class B (VIM enzyme)					
Ambler Class C (AmpC enzyme)					
Ambler Class D (OXA enzyme)					
Sample collection	Rectal swab, 2 h after ICU admission, prior to antimicrobial therapy	Rectal swab, ICU admission + weekly	Rectal swab, ICU admission + weekly + discharge	Rectal swab, ICU admission, prior study intervention + days 3, 7, 14, 30	Stool sample, after written informed consent
Microbiota sequence processing	PCR amplification V3–V4 regions 16S rRNA + ITS2 loci of rDNA, sequencing: Illumina, pipeline: DADA2	PCR amplification V4 region 16S rRNA, sequencing: Illumina, pipeline: Mothur	PCR amplification V4 region 16S rRNA, sequencing: Illumina, pipeline: USEARCH	PCR amplification V3–V4 region 16S rRNA, sequencing: Illumina, pipeline: DADA2	PCR amplification V3–V4 region 16S rRNA, sequencing: Illumina, pipeline: EzBioCloud
Microbiota α‐diversity metrics	Shannon, Simpson, evenness	invSimpson	Shannon, Simpson, invSimpson, Jost1	Shannon, Chao1	Shannon, Chao1, OTUs
Microbiota β‐diversity metrics	Bray–Curtis dissimilarity	Yue and Clayton θ dissimilarity index		UniFrac distance	Bray–Curtis dissimilarity

Study name	García‐García	Ling	Sindi	Fontaine	Rubio Garcia
Year	2023	2025	2022	2020	2022
Region, country	Europe, Spain	East Asia, China	West Asia, Saudi Arabia	Europe, France	Europe, Spain
Study design	Prospective	Prospective	Prospective	Prospective	Prospective
Population enrollment period	March–May 2021	April–December 2024	Not specified	January–March 2018	July–December 2018
Inclusion and exclusion criteria	I: > 18 yo ICU patients with an ICU stay > 48 h with SarsCov2 infection	I: Adult ICU patients	I: Adult ICU patients	I: Adult ICU patients with mechanical ventilation at admission predicted to last > 3 days, spontaneously passing feces	I: Adult ICU patients
Comparison case/control	MDROs‐carriers (8)/noncarriers (9)	MDROs‐carriers (100)/matched control (86)	CRE‐carriers (24)/noncarriers (26)/matched control (10)	MDROs‐carriers (13)/noncarriers (18)	At‐admission MDROs‐carriers (19)/during‐stay MDROs‐carriers (16)/control (27)
Control definition	Noncarriers ICU patients	Healthy individuals, matched on gender and age, screened through telephone and in‐person, excluding those with recent hospitalization, antibiotic use, or confirmed MDROs‐exposure	(1) Noncarriers ICU patients; (2) healthy adults, matched on age, without antibiotic treatment for 6 months, or gastrointestinal disease prior to sampling	Noncarriers ICU patients	ICU patients who were not colonized at ICU admission and did not become colonized during ICU stay
Age (years), mean (SD)/median (IQR) [range] case/control	67 (65–69)/64 (58–67)	Carriers/control: 60.05 ± 18.53/58.83 ± 18.78	Carriers/noncarriers: 61.8 ± 3.5 (23–89)/59.9 ± 3.4 (18–88); carriers/control: 61.8 ± 3.5 (23–89)/58 ± 8.2 (20–90)		At‐admission carriers/noncarriers: 72 (67–75.5)/63 (49.5–72); during‐stay carriers/noncarriers: 59.5 (51–69.75)/63 (49.5–72)
Female (%) case/control	25%/45%	34%/37.2%	Carriers/noncarriers: 50%/65.38%; carriers/control: 50%/40%		At‐admission carriers/noncarriers: 15.79%/40.74%; during‐stay carriers/noncarriers: 50%/40.74%
Race (%) case/control					
BMI (kg/m^2^), mean (SD)/median (IQR) [range] case/control		23.81 ± 6.43/24.96 ± 5.95			
Comorbidity index (score), mean (SD)/median (IQR) [range] case/control					Charlson: At‐admission carriers/noncarriers 7 (6–8)/4 (3–6); during‐stay carriers/noncarriers: 4.5 (4–5.25)/4 (3–6)
Hospitalization history (%) case/control					At‐admission carriers/noncarriers: 42.11%/33.33%; during‐stay carriers/noncarriers: 62.5%/33.33%
Antibiotic therapy (%) case/control					
Type of ICU	Medical	Not specified	Medical	Medical	Medical
ICU Admission Severity Scoring Systems (score), mean (SD)/median (IQR) [range] case/control	SOFA: 7 (6–9)/7 (6–8); APACHE: 17 ± 4/15 ± 3				
Cause of ICU admission (%) case/control					
H2 blocker or proton pump inhibitor (%) case/control					
Opioids (%) case/control					
Steroids (%) case					
Vasoactive agent (%) case/control	75%/90%				
Antibiotic (%) case/control	75%/44%	85%/0%			
β‐Lactam class (%) case/control					
Quinolone class (%) case/control					
Piperacillin‐tazobactam class (%) case/control					
Macrolide class (%) case/control					
Vancomycin class (%) case/control					
Carbapenem class (%) case/control					
Artificial nutrition (%) case/control					
Infection (%) case/control			Bacteremia 37.5%/30.80%		At‐admission carriers/noncarriers: 68.42%/70.37%; during‐stay carriers/noncarriers: 81.25%/70.37%
Mechanical ventilation (%) case/control	100%/100%				At‐admission carriers/noncarriers: 15.79%/25.93%; during‐stay carriers/noncarriers: 43.75%/25.93%
Renal replacement therapy (%) case/control	25%/11%				At‐admission carriers/noncarriers: 15.79%/7.41%; during‐stay carriers/noncarriers: 12.5%/7.41%
Length of ICU stay (days), mean (SD)/median (IQR) [range] case/control	31 ± 22/36 ± 23		29 ± 5/11.5 ± 2.5		At‐admission carriers/noncarriers: 6 (4–12.5)/7 (4–9.5); during‐stay carriers/noncarriers: 8.5 (5.75–23)/7 (4–9.5)
Length of hospital stay (days), mean (SD)/median (IQR) [range] case/control					At‐admission carriers/noncarriers: 18 (11.5–51)/19 (13–40.5); during‐stay carriers/noncarriers: 30 (14–48.75)/19 (13–40.5)
ICU‐mortality (%) case/control	38%/11%		62.5%/46.15%		At‐admission carriers/noncarriers: 15.79%/11.11%; during‐stay carriers/noncarriers: 12.5%/11.11%
In‐hospital mortality (%) case/control					At‐admission carriers/noncarriers: 42.11%/18.52%; during‐stay carriers/noncarriers: 31.25%/18.52%
AmpC β‐lactamase–producing‐E				4	10
ESBL‐E	1			14	20
CRE	6		24		7
VRE					
Nonfermenter GNB	2			1	10
AmpC β‐lactamase‐*H. alvei*				1	
AmpC β‐lactamase‐*M. morganii*				1	1
AmpC β‐lactamase‐*E. aerogenes*				1	1
AmpC β‐lactamase‐*E. cloacae*				1	6
AmpC β‐lactamase‐*C. freundii*					1
AmpC β‐lactamase‐*C. farmeri*					1
ESBL‐*K. pneumoniae*				4	11
ESBL‐*E. coli*	1			9	9
ESBL‐*C. freundii*				1	
CR‐*K. pneumoniae*	1				5
CR‐*E. coli*					1
CR‐*K. oxytoca*					1
CR‐*E. cloacae*	4				
CR‐*S. marcescens*	1				
CR‐*P. aeruginosa*					
NFGNB‐*S. maltophilia*	1			1	4
NFGNB‐*P. aeruginosa*	1				6
Ambler Class A (KPC enzyme)					2
Ambler Class B (VIM enzyme)	2				1
Ambler Class C (AmpC enzyme)				4	10
Ambler Class D (OXA enzyme)	6				4
Sample collection	Rectal swab, ICU admission	Stool sample	Stool sample, during ICU hospitalization	Stool sample + endotracheal aspiration, admission + twice a week	Rectal swab, ICU admission + weekly
Microbiota sequence processing	PCR amplification V4‐V5 region 16S rRNA, sequencing: Illumina, pipeline: QIIME2	PCR amplification V3–V4 regions 16S rRNA, sequencing: Illumina, pipeline: QIIME2	PCR amplification V3, V4, V3–V4 regions 16S rRNA, sequencing: Illumina, pipeline: QIIME 1.9.1	PCR amplification V4 region 16S rRNA, sequencing: Illumina, pipeline: Shaman	PCR amplification V3–V4 region 16S rRNA gene, sequencing: Illumina, pipeline: QIIME2
Microbiota α‐diversity metrics	Shannon, invSimpson, observed species	Shannon, Simpson, Chao1, observed species, ACE	Shannon, observed species	Shannon, OTUs	Shannon, OTUs, Faith's phylogenetic diversity, Pielou's index
Microbiota β‐diversity metrics	Bray–Curtis dissimilarity, unweighted and weighted UniFrac distance	Bray–Curtis dissimilarity, unweighted and weighted UniFrac distance	Aitchison distance		

Abbreviations: ACE, abundance‐based coverage estimator; AmpC β‐lactamase–producing‐E, AmpC β‐lactamase–producing‐Enterobacterales; APACHE II, Acute Physiology and Chronic Health Evaluation II; ARDS, acute respiratory distress syndrome; BMI, body mass index; CR, carbapenem resistant; *CRE*, carbapenem‐resistant Enterobacterales; CRPA, *carbapenem‐resistant Pseudomonas aeruginosa*; DADA2, deficiency of adenosine deaminase; E, exclusion; *E. coli*, *Escherichia coli*; ESBL‐E, extended‐spectrum β‐lactamase–producing Enterobacterales; I, inclusion; ICU, intensive care unit; IQR, interquartile range; ITS2, internal transcribed spacer 2; *K. oxytoca*, *Klebsiella oxytoca*; *K. pneumoniae*, *Klebsiella pneumoniae*; KPC, *Klebsiella pneumoniae* carbapenemase; MDROs, multidrug‐resistant organisms; NFGNB, non‐fermentative Gram‐negative Bacillus; Nonfermenter GNB, nonfermenter Gram‐negative bacteria; OTU, operational taxonomic unit; OXA, oxacillinases; *P. aeruginosa*, *Pseudomonas aeruginosa*; PCR, polymerase chain reaction; PPI, proton pump inhibitor; rDNA, ribosomal DNA; rRNA, ribosomal ribonucleic acid; SAPS II, Simplified Acute Physiology Score II; SOFA, Sequential Organ Failure Assessment; VIM, Verona Integron‐encoded Metallo‐β‐lactamase; VRE, vancomycin‐resistant *Enterococcus*; yo, years old.

See Tables A10a and A10b.

**Table A2:** Comparison of Shannon index and Simpson's index between VRE‐colonized ICU patients and noncolonized patients.

Characteristic	*N* studies	*N* VRE‐colonized	*N* noncolonized	MD (95% CI)	*p* Value	*I* ^2^ (95% CI)	*p* Value	Studies
Shannon index	1	18	72	−0.75 (−1.38 to 2)	0.02	NC		Park et al. (2025)
Simpson's index	1	175	1977	−1.1 (−1.92 to −0.28)	0.009	NC		Collingwood et al. (2020)

Abbreviations: CI, confidence interval; *I* ^2^, the percentage of variance attributable to study heterogeneity; ICU, intensive care unit; MD, mean difference; *N*, number of participants; NC, cannot be computed due to the small number of studies; VRE, vancomycin‐resistant *Enterococcus*.

**Table A3:** Comparison of microbiota structure and composition between CRE‐colonized and noncolonized ICU patients.

Characteristic, effect size type	*N* studies	*N* CRE‐colonized	*N* noncolonized	MD (95% CI)	*p* Value	*I* ^2^ (95% CI)	*p* Value	Studies
Shannon index	3	74	103	−0.1 (−0.37 to 0.17)	0.483	29.3 (0–92.6)	0.243	Pettigrew et al. (2019), Sindi et al. (2022), and Baek et al. (2023)
Simpson's index	1	41	45	−0.03 (−0.16 to 0.1)	0.645	NC		Pettigrew et al. (2019)
invSimpson index	1	41	45	0.93 (−0.92 to 2.78)	0.323	NC		Pettigrew et al. (2019)
OTU	2	33	58	−167.71 (−522.23 to 186.81)	0.354	92.4 (74.1–97.8)	< 0.001	Sindi et al. (2022) and Baek et al. (2023)
Relative abundance, Pseudomonadota phylum	2	33	58	0.21 (0.04–0.39)	0.018	29.9 (NA–NA)	0.232	Sindi et al. (2022) and Baek et al. (2023)
Relative abundance, Bacteroidetes phylum	2	33	58	−0.2 (−0.63 to 0.23)	0.369	91.8 (71.5–97.6)	< 0.001	Sindi et al. (2022) and Baek et al. (2023)
Relative abundance, Bacillota phylum	2	33	58	−0.09 (−0.24 to 0.06)	0.247	0 (NA–NA)	0.799	Sindi et al. (2022) and Baek et al. (2023)
Relative abundance, Bifidobacteriaceae family	1	24	26	−0.11 (−0.19 to −0.02)	0.012	NC		Sindi et al. (2022)
Relative abundance, Coriobacteriaceae family	1	24	26	0 (−0.01 to 0)	0.046	NC		Sindi et al. (2022)
Relative abundance, Enterobacteriaceae family	1	24	26	0.37 (0.16–0.58)	< 0.001	NC		Sindi et al. (2022)

Abbreviations: CI, confidence interval; CRE, carbapenem‐resistant Enterobacterales; *I* ^2^, the percentage of variance attributable to study heterogeneity; ICU, intensive care unit; MD, mean difference; *N*, number of participants; NC, cannot be computed due to the small number of studies; OTU, operational taxonomic unit.

**Table A4:** Comparison of microbiota structure and composition between CRE‐colonized ICU patients and control patients.

Characteristic	*N* studies	*N* CRE	*N* control	MD (95% CI)	*p* Value	*I* ^2^ (95% CI)	*p* Value	Studies	
Shannon index	2	65	40	−1.09 (−2.18 to 0)	0.049	94.2 (81.7–98.2)	< 0.001	NC	Pettigrew et al. (2019) and Sindi et al. (2022)
Simpson's index	1	41	30	0.11 (−0.02 to 0.24)	0.098	NC		NC	Pettigrew et al. (2019)
invSimpson index	1	41	30	−0.98 (−3.52 to 1.56)	0.45	NC		NC	Pettigrew et al. (2019)
OTU	1	24	10	−1.63 (−1.89 to −1.37)	< 0.001	NC		NC	Sindi et al. (2022)
Relative abundance, Pseudomonadota Phylum	1	24	10	0.21 (0.09–0.33)	< 0.001	NC		NC	Sindi et al. (2022)
Relative abundance, Bacteroidetes phylum	1	24	10	0.02 (−0.1 to 0.14)	0.733	NC		NC	Sindi et al. (2022)
Relative abundance, Bacillota phylum	1	24	10	−0.1 (−0.29 to 0.09)	0.305	NC		NC	Sindi et al. (2022)
Relative abundance, Bifidobacteriaceae family	1	24	10	−0.51 (−0.91 to −0.12)	0.011	NC		NC	Sindi et al. (2022)
Relative abundance, Coriobacteriaceae Family	1	24	10	−0.1 (−0.19 to 0)	0.045	NC		NC	Sindi et al. (2022)
Relative abundance, Enterobacteriaceae family	1	24	10	0.42 (0.22–0.63)	< 0.001	NC		NC	Sindi et al. (2022)

Abbreviations: CI, confidence interval; CRE, carbapenem‐resistant Enterobacterales; *I* ^2^, the percentage of variance attributable to study heterogeneity; ICU, intensive care unit; MD, mean difference; *N*, number of participants; NC, cannot be computed due to the small number of studies; OTU, operational taxonomic unit.

**Table A5:** Taxa positively associated with multidrug‐resistant‐organisms‐colonization in critically ill patients.

Multidrug‐resistant organisms	Association	Species	Genus	Family	Order	Phylum	Method	Author
Extended‐spectrum β‐lactamase–producing *Escherichia coli*	Abundant	*Candida albicans*	—	—	—	—	LDA LEfSe, LDA score > 3 log	Prevel et al.
Extended‐spectrum β‐lactamase–producing *Klebsiella pneumoniae*	Abundant	*Issatchenkia orientalis* (*Candida krusei*), *Dipodascus geotrichum, Candida tropicalis*	*Saccharomyces*	—	—	—	LDA LEfSe, LDA score > 3 log	Prevel et al.
Vancomycin‐resistant *Enterococcus*	Predictive	*Bacteroides caccae, Lactobacillus gasseri, Clostridium incertae sedis, Erysipelatoclostridium ramosum, Bacteroides vulgatus, Bacteroides tetaiotathetamicron*	*Enterococcus*	—	—	—	Random Forest analysis	Park et al.
	Abundant	—	*Enterococcus*	—	—	—	Nonparametric Kruskal–Wallis test	Collingwood et al.
	Abundant	*Bacteroides thetaiotamicron, Bacteroides vulgatus*	*Enterococcus*	—	—	—	ANCOM2	Park et al.
Carbapenem‐resistant Enterobacterales	Abundant	—	*Erwinia, Citrobacter, Klebsiella, Cronobacter, Kluyvera, Dysgonomonas, Pantoea, Alistipes*	Enterobacteriaceae, Rikenellaceae, Erwiniaceae	—	Proteobacteria	LDA LEfSe, LDA score > 2 log	Baek et al.
	Abundant	—	*Enterococcus, Sphingomonas, Staphylococcus, Klebsiella, Parabacteroides, Proteus, Pseudomonas*	Enterobacteriaceae, Morganellaceae, Pseudomonadaceae	—	Proteobacteria	Pairwise differential abundance	Sindi et al.
Multidrug‐resistant organisms	Predictive	—	*Enterococcus, Klebsiella, Roseburia, Pseudomonas, Bacteroides, Faecalibacterium*	—	—	—	Random Forest analysis	Ling et al.
	Abundant	—	*Parabacteroides, Klebsiella, Clostridioides, Enterococcus, Corynebacterium, Escherichia‐Shigella, Pelomonas, Eubacterium_coprostanoligenes_group*	—	—	—	LDA LEfSe, LDA score > 3 log	Ling et al.
	Abundant	—	*Enterococcus, Parabacteroides, Klebsiella, Clostridioides*	Enterobacteriaceae, Enterococcacceae	—	Proteobacteria	MetaStats 2.0	Ling et. al
	Abundant	—	*Klebsiella*	Enterobacteriaceae	—	—	LDA LEfSe, LDA score = 4	Rubio Garcia et al.
	Abundant	*Dialister propionicifaciens*	—	—	—	—	LDA LEfSe, LDA score = 2 log	Garcia‐Garcia et al.
	Abundant	—	*Anaerococcus, Dialister, Peptoniphilus*	—	—	—	Differential abundance analysis	Garcia‐Garcia et al.
Number of unique taxa	—	12	26	6	—	—	—	—
Most reported	—	—	*Enterococcus, Klebsiella*	—	—	—	—	—

Abbreviations: LDA, linear discriminant analysis; LEfSe, Linear discriminant analysis Effect Size; ANCOM2, analysis of compositions of microbiomes 2.

**Table A6:** Taxa negatively associated with multidrug‐resistant‐colonization in critically ill patients.

Multidrug‐resistant organisms	Association	Species	Genus	Family	Order	Phylum	Method	Author
Carbapenem‐resistant *Pseudomonas aeruginosa*	Reduced odds	—	*Peptoniphilus, Prevotella, Mogibacterium, Varibaculum, Dialister, Ezakiella, Porphyromonas, Finegoldia, Murdochiella*	—	Clostridiales, Actinomycetales	—	PCA + logistic regression	Pettigrew et al.
Carbapenem‐resistant Enterobacterales	Depleted	—	*Barnesiella, Subdoligranulum, Eisenbergiella, Frisingicoccus, Longicatena, Roseburia, Oscillibacter, Parabacteroides, Romboutsia, Bacteroides, Mogibacterium*	Barnesiellaceae, Mogibacterium, Porphyromonadaceae, Bacteroidaceae	—	Bacteroidetes	LDA LEfSe, LDA < 2 log	Baek et al.
	Depleted	—	*Anaerostipes, Blautia, Collinsella, Dialister, Eubacterium, Faecalibacterium, Prevotella, Roseburia, Bifidobacterium, Helicobacter, Lachnospira, Romboutsia, Turicibacter*	Corynebacteriaceae, Sphingomonadaceae	—	—	Pairwise differential abundance	Sindi et al.
Multidrug‐resistant organisms	Depleted	—	*Bacteroides, Prevotella_9, Faecalibacterium, Oscillospiraceae_ UCG‐002, Roseburia, Megasphaera, Sutterella, Agathobacter, Anaerostipes, Eubacterium_hallii_group, Eubacterium_eligens_group, Coprococcus*	Bacteroidaceae, Lachnospiraceae	—	Bacteroidota	MetaStats 2.0	Ling et al.
Number of unique taxa	—	—	36	7	—	—	—	—
Most reported	—	—	*Prevotella, Roseburia*	—	—	—	—	—

Abbreviations: LDA, linear discriminant analysis; LEfSe, Linear discriminant analysis Effect Size; PCA, principal component analysis.

**Table A7:** Taxa positively and negatively associated with infection in critically ill patients.

Association	Species	Genus	Family	Order	Phylum	Method	Author
Positive/abundant	—	*Pseudomonas, Providencia, Klebsiella*	—	—	—	LDA LefSe, LDA score > 3 log	Ling et al.
Positive/abundant	—	*Shigella‐Escherichia, Pseudomonas, Enterococcus*	Enterococcaceae, Pseudomonadaceae	—	—	LDA LEfSe, LDA score = 4 log	Rubio Garcia et al.
Positive/predictive	—	*Enterococcus, Klebsiella, Roseburia, Pseudomonas, Bacteroides, Faecalibacterium, Escherichia‐Shigella, Klebsiella, Pelomonas, Eubacterium coprostanoligenes group, Pseudomonas, Lactobacillus, Providencia*	—	—	—	Random Forest analysis	Ling et al.
Negative/depleted	—	*Finegoldia, Prevotella, Peptoniphilus, Anaerococcus, Paraprevotella, Coprobacter*	Family XI, Prevotellaceae	—	—	LDA LEfSe, LDA = 4	Rubio Garcia et al.
Negative/depleted	—	*Bacteroides, Alistipes, Lactobacillus*	—	—	—	MetaStats2.0	Ling et al.
Negative/reduced odds	—	—	Family XI	—	—	LASSO regression	Rubio Garcia et al.

Abbreviations: LDA, linear discriminant analysis; LEfSe, Linear discriminant analysis Effect Size; LASSO, least absolute shrinkage and selection operator.

**Table A8:** Taxa positively and negatively associated with mortality in critically ill patients.

Association	Specie	Genus	Family	Order	Phylum	Method	Author
Positive/abundant	—	*Enterococcus, Coprobacter, Pseudomonas*	Enterococcaceae, Pseudomonadaceae	Lactobacillales, Pseudomonadales	—	LDA LEfSe, LDA score = 4	Rubio Garcia et al.
Negative/depleted	—	*Prevotella*	Family XI, Prevotellaceae	Bacteroidales, Clostridiales	Bacteroidetes	LDA LEfSe, LDA score = 4	Rubio Garcia et al.
Negative/reduced mortality	—	*Corynebacterium*	—	—	—	LEfSe	Garcia‐Garcia et al.

Abbreviations: LDA, linear discriminant analysis; LEfSe, Linear discriminant analysis Effect Size.

**Table A9:** Taxa positively associated with the absence of multidrug‐resistant‐organisms‐colonization.

Context	Species	Genus	Family	Order	Phylum	Method	Author
Critically ill, noncolonized	*Candida albicans*	—	—	—	—	LDA LEfSe, LDA score > 3 log	Prevel et al.
Critically ill, noncolonized	*Campylobacter hominis, Campylobacter ureolyticus, Dialister invisus*	*Porphyromonas, Peptoniphilus, Anaerococcus, Saccharomyces*	—	—	—	LDA LEfSe, LDA score > 3 log	Prevel et al.
Critically ill, noncolonized	—	*Pseudomonal* spp.	—	—	—	Abundance of taxa from 16S sequencing	Park et. al
Critically ill, noncolonized	*Varibaculum cambriense, Citrobacter europaeus, Proteus bacterium_R49*	—	—	—	—	LDA LEfSe, LDA score = 2 log	Garcia‐Garcia et al.
Critically ill, noncolonized	—	*Enterococcus, Ochrobactrum, Staphylococcus*	—	—	—	Differential abundance analysis	Garcia‐Garcia et al.
Critically ill, noncolonized	—	*Prevotella, Finegoldia*	Family XI, Prevotellaceae		—	LDA LEfSe, LDA score = 4	Rubio Garcia et al.
Healthy	—	*Blautia, Faecalibacterium, Roseburia, Bifidobacterium, Prevotella, Bacteroides*	—	—		LEfSe	Ling et al.
Healthy	—	—	Bifidobacteriaceae, Succinivibrionaceae, Coriobacteriaceae	—	Actinobacteria	Pairwise differential abundance	Sindi et al.
Number of unique taxa	7	15	5	—	—	—	—
Most reported	—	*Prevotella*		—	—	—	—

Abbreviations: LDA, linear discriminant analysis; LEfSe, Linear discriminant analysis Effect Size.

**Table A10a:** STORMS checklist, part 1.

Version	1.03				Pettigrew, 08/2019		Collingwood, 01/2020		Fontaine, 08/2020		Sindi, 06/2022		Rubio Garcia, 07/2022	
Number	Item	Recommendation	Item source	Additional guidance	Yes/No/NA	Comments or location in manuscript	Yes/No/NA	Comments or location in manuscript	Yes/No/NA	Comments or location in manuscript	Yes/No/NA	Comments or location in manuscript	Yes/No/NA	Comments or location in manuscript
*Abstract*														
1.0	Structured or unstructured abstract	Abstract should include information on background, methods, results, and conclusions in a structured or unstructured format.	STORMS		Yes	Abstract	Yes	Abstract	Yes	Abstract	Yes	Abstract	Yes	Abstract
1.1	Study design	State study design in abstract.	STORMS	See 3.0 for additional information on study design.	No	Partial	No	Not reported	Yes	Abstract—methods	No	Partial	Yes	Abstract—methods
1.2	Sequencing methods	State the strategy used for metagenomic classification.	STORMS	For example, targeted 16S by qPCR or sequencing, shotgun metagenomics, metatranscriptomics, and so forth.	Yes	Abstract—methods	Yes	Abstract—methods	Yes	Abstract—results	Yes	Abstract	Yes	Abstract—methods
1.3	Specimens	Describe body site(s) studied.	STORMS		No	Not reported	Yes	Abstract—methods	Yes	Abstract—methods	Yes	Abstract	Yes	Abstract—methods
*Introduction*														
2.0	Background and Rationale	Summarize the underlying background, scientific evidence, or theory driving the current hypothesis, as well as the study objectives.	STORMS		Yes	Introduction	Yes	Introduction	Yes	Introduction	Yes	Introduction	Yes	Introduction
2.1	Hypotheses	State the prespecified hypothesis. If the study is exploratory, state any prespecified study objectives.	STORMS		Yes	Introduction	Yes	Introduction	Yes	Introduction	Yes	Introduction	Yes	Introduction
*Methods*														
3.0	Study design	Describe the study design.	STORMS	Observational (case–control, cohort, cross‐sectional survey, etc.) or experimental (randomized controlled trial, nonrandomized controlled trial, etc.). For a brief description of common study designs, see: DOI: 10.11613/BM.2014.022 If applicable, describe any blinding (e.g., single or double‐blinding) used in the course of the study.	Yes	Methods—study design and participants	No	Not reported	Yes	Material and methods	No	Not reported	Yes	Methods—study design and clinical definitions
3.1	Participants	State what the population of interest is, and the method by which participants are sampled from that population. Include relevant information on the physiological state of the subjects or the stage in the life history of the disease under study when participants were sampled.	STORMS	Examples of the population of interest could be: adults with no chronic health conditions, adults with type II diabetes, newborns, and so forth. This is the total population to which the study is hoped to be generalizable. The sampling method describes how potential participants were selected from that population. If the participants are from a substudy of a larger study, provide a brief description of the larger study and cite it. Clearly state how cases and controls are defined. An example of a relevant physiological state might be pre/post menopausal for a vaginal microbiome study; examples of stages in the life history of disease could be whether specimens were collected during active or dormant disease, or before or after treatment.	Yes	Methods—study design and participants	Yes	Methods—bacterial identification and patient population	Yes	Material and methods—population	Yes	Materials and methods—study design and sample collection	Yes	Methods—study design and clinical definitions
3.2	Geographic location	State the geographic region(s) where participants were sampled from.	MIxS: geographic location (country and/or sea, region)	Geographic coordinates can be reported to prevent potential ambiguities if necessary.	Yes	Methods—study design and participants	Yes	Methods—sample collection	Yes	Material and methods	Yes	Materials and methods—study design and sample collection	Yes	Methods—study design and clinical definitions
3.3	Relevant dates	State the start and end dates for recruitment, follow‐up, and data collection.	STORMS	Recruitment is the period in which participants are recruited for the study. In longitudinal studies, follow‐up is the time period during which participants are asked to complete a specific assessment. Finally, data collection is the total period in which data is being collected from participants, including during initial recruitment through all follow‐ups.	Yes	Methods—study design and participants	Yes	Methods—sample collection	Yes	Material and methods—population	No	Not reported	Yes	Methods—study design and clinical definitions
3.4	Eligibility criteria	List any criteria for inclusion and exclusion of recruited participants.	Modified STROBE	Among potential recruited participants, how were some chosen and others not? This could include criteria, such as sex, diet, age, health status, or BMI. If there is a primary and validation sample, describe the inclusion/exclusion criteria for each.	Yes	Methods—study design and participants	Yes	Methods—bacterial identification and patient population	Yes	Material and methods—population	No	Partial	Yes	Methods—study design and clinical definitions
3.5	Antibiotics Usage	List what is known about antibiotic usage before or during sample collection.	STORMS	If participants were excluded due to current or recent antibiotic usage, state this here. Other factors (e.g., proton pump inhibitors, probiotics, etc.) that may influence the microbiome should also be described as well.	Yes	Methods—study design and participants	Yes	Methods—demographic definitions	No	Not reported	No	Partial	No	Not reported
3.6	Analytic sample size	Explain how the final analytic sample size was calculated, including the number of cases and controls if relevant, and reasons for dropout at each stage of the study. This should include the number of individuals in whom microbiome sequencing was attempted and the number in whom microbiome sequencing was successful.	STORMS	Consider use of a flow diagram (see template at https://stormsmicrobiome.org/figures). Also state the sample size in the Abstract. If power analysis was used to calculate sample size, describe those calculations.	No	Not reported	Yes	Methods—bacterial identification and patient population	No	Not reported	No	Not reported	No	Not reported
3.7	Longitudinal studies	For longitudinal studies, state how many follow‐ups were conducted, describe the sample size at follow‐up by group or condition, and discuss any loss to follow‐up.	STORMS	If there is loss to follow‐up, discuss the likelihood that drop‐out is associated with exposures, treatments, or outcomes of interest.	Yes	Methods—study design and participants	NA		Yes	Material and methods—sample collection	NA		Yes	Methods—study design and clinical definitions
3.8	Matching	For matched studies, give matching criteria.	Modified STROBE	“Matched” refers to matching between comparable study participants as cases and controls or exposed/unexposed. Indicate whether participants were individually or frequency matched and in what ratio they were matched (e.g., 1 case to 1 control).	No	Methods—study design and participants	No	Not reported	No	Not reported	Yes	Healthy controls matched by age	No	Not reported
3.9	Ethics	State the name of the institutional review board that approved the study and protocols, protocol number and date of approval, and procedures for obtaining informed consent from participants.	STORMS		No	Partial, methods—study design and participants	Yes	Methods—sample collection	Yes	Material and methods—population	No	Partial	Yes	Methods—ethics approval
4.0	Laboratory methods	State the laboratory/center where laboratory work was done.	STORMS	Provide a reference to complete lab protocols if previously published elsewhere, such as on protocols.io. Note any modifications of lab protocols and the reason for protocol modifications.	Yes	Methods—sequencing and analysis of 16S ribosomal RNA gene sequences	Yes	Methods—DNA purification and microbiota analysis	Yes	Material and methods—sample collection	No	Not reported	Yes	Methods—16s rRNA gene sequencing and bioinformatic approach
4.1	Specimen collection	State the body site(s) sampled from and how specimens were collected.	MIxS: sample collection device or method; host body site	Use terms from the Uber‐anatomy Ontology (https://www.ebi.ac.uk/ols/ontologies/uberon) to describe body sites in a standardized format.	Yes	Methods—study design and participants	Yes	Methods—sample collection	Yes	Material and methods—sample collection	Yes	Materials and methods—study design and sample collection	Yes	Abstract—study design and clinical definitions
4.2	Shipping	Describe how samples were stored and shipped to the laboratory.	STORMS	Include the length of time from collection to receipt by the lab, and if temperature control was used during shipping.	No	Not reported	No	Not reported	Yes	Material and methods—sample collection	No	Not reported	No	Not reported
4.3	Storage	Describe how the laboratory stored samples, including the time between collection and storage and any preservation buffers or refrigeration used.	STORMS	State where each procedure or lot of samples was done, if not all in the same place. Include reagent/lot/catalog #s for storage buffers.	Yes	Methods—study design and participants	Yes	Methods—bacterial identification and patient population	Yes	Material and methods—sample collection	No	Partial	No	Partial
4.4	DNA extraction	Provide the DNA extraction method, including the kit and version, if relevant.	MIxS: nucleic acid extraction	If any DNA quantification methods were used prior to DNA amplification or at the pooling step of library preparation, state so here.	Yes	Methods—sequencing and analysis of 16S ribosomal RNA gene sequences	No	Methods—DNA purification and microbiota analysis	Yes	Material and methods—DNA extraction 16S RNA gene sequencing	Yes	Materials and methods—microbiota sequencing and taxonomy assignment	Yes	Methods—16s rRNA gene sequencing and bioinformatic approach
4.5	Human DNA sequence depletion or microbial DNA enrichment	Describe whether human DNA sequence depletion or enrichment of microbial or viral DNA was performed.	STORMS		No	Not reported	No	Not reported	No	Not reported	No	Not reported	No	Not reported
4.6	Primer selection	Provide primer selection and DNA amplification methods, as well as variable region sequencing (if applicable).	MIxS: PCR primers		Yes	Methods—sequencing and analysis of 16S ribosomal RNA gene sequences	Yes	Methods—DNA purification and microbiota analysis	Yes	Material and methods—16S RNA gene sequencing	Yes	Materials and methods—microbiota sequencing and taxonomy assignment	Yes	Methods—16s rRNA gene sequencing and bioinformatic approach
4.7	Positive controls	Describe any positive controls (mock communities) if used.	STORMS	If used, it should be deposited under the guidance provided in the 8.X items.	No	Not reported	No	Not reported	No	Not reported	No	Not reported	No	Partial
4.8	Negative controls	Describe any negative controls if used.	STORMS	If used, it should be deposited under the guidance provided in the 8.X items.	Yes	Methods—sequencing and analysis of 16S ribosomal RNA gene sequences	No	Not reported	No	Not reported	No	Not reported	No	Partial
4.9	Contaminant mitigation and identification	Provide any laboratory or computational methods used to control for or identify microbiome contamination from the environment, reagents, or laboratory.	STORMS	Includes filtering of reagents and other steps to minimize contamination. It is relevant to state whether the specimens of interest have low microbial load, which makes contamination especially relevant.	No	Not reported	No	Not reported	No	Not reported	No	Not reported	No	Partial
4.10	Replication	Describe any biological or technical replicates included in the sequencing, including which steps were replicated between them.	STORMS	Replication may be biological (redundant biological specimens) or technical (aliquots taken at different stages of analysis) and used in extraction, sequencing, preprocessing, and/or data analysis.	Yes	Methods—sequencing and analysis of 16S ribosomal RNA gene sequences	No	Not reported	No	Not reported	No	Not reported	No	Partial
4.11	Sequencing strategy	Major strategy divisions, such as shotgun or amplicon sequencing.	MIxS: sequencing method	For amplicon sequencing (e.g., 16S variable region), state the region selected. State the model of the sequencer used.	Yes	Methods—sequencing and analysis of 16S ribosomal RNA gene sequences	Yes	Methods—DNA purification and microbiota analysis	Yes	Material and methods—16S RNA gene sequencing	Yes	Materials and methods—microbiota sequencing and taxonomy assignment	Yes	Methods—16s rRNA gene sequencing and bioinformatic approach
4.12	Sequencing methods	State whether experimental quantification was used (QMP/cell count‐based, spike‐in‐based) or whether relative abundance methods were applied.	STORMS	These include read length, sequencing depth per sample (average and minimum), whether reads are paired, and other parameters.	Yes	Methods—sequencing and analysis of 16S ribosomal RNA gene sequences	Yes	Methods—DNA purification and microbiota analysis	Yes	Material and methods—bioinformatic analyses	No	Partial	Yes	Methods—16s rRNA gene sequencing and bioinformatic approach
4.13	Batch effects	Detail any blocking or randomization used in study design to avoid confounding of batches with exposures or outcomes. Discuss any likely sources of batch effects, if known.	STORMS	Sources of batch effects include sample collection, storage, library preparation, and sequencing and are commonly unavoidable in all but the smallest of studies.	No	Not reported	No	Not reported	No	Not reported	No	Not reported	No	Partial
4.14	Metatranscriptomics	Detail whether any mRNA enrichment was performed and whether/how retrotranscription was performed prior to sequencing. Provide a size range of isolated transcripts. Describe whether the sequencing library was stranded or not. Provide details on sequencing methods and platforms.	STORMS	Provide details on any internal standards which may have been used, as well as parameters and versions of any software or databases used.	NA	Not reported	NA		NA		NA		NA	
4.15	Metaproteomics	Detail which protease was used for digestion. Provide details on proteomic methods and platforms (e.g., LC‐MS/MS, instrument type, column type, mass range, resolution, scan speed, maximum injection time, isolation window, normalized collision energy, and resolution).	STORMS	Provide details on any internal standards which may have been used, as well as parameters and versions of any software or databases used.	NA	Not reported	NA		NA		NA		NA	
4.16	Metabolomics	Specify the analytical method used (e.g., nuclear magnetic resonance spectroscopy or mass spectrometry). For mass spectrometry, provide details on which fractions were obtained (polar and/or nonpolar) and how these were analyzed. Provide details on metabolomics methods and platforms (e.g., derivatization, instrument type, injection type, column type, and instrument settings).	STORMS	Provide details on any internal standards which may have been used, as well as parameters and versions of any software or databases used.	NA	Not reported	NA		NA		NA		NA	
5.0	Data sources/measurement	For each nonmicrobiome variable, including the health condition, intervention, or other variable of interest, state how it was defined, how it was measured or collected, and any transformations applied to the variable prior to analysis.	MIxS: host disease status	State any sources of potential bias in measurements, for example, multiple interviewers or measurement instruments, and whether these potential biases were assessed or accounted for in the study design. Use terms from a standardized ontology, such as the Experimental Factor Ontology (https://www.ebi.ac.uk/efo/), to describe variables of interest in a standardized format.	Yes	Methods—study design and participants	No	Methods—demographic definitions	Yes	Material and methods	No	Partial	Yes	Methods—study design and clinical definitions
6.0	Research design for causal inference	Discuss any potential for confounding by variables that may influence both the outcome and exposure of interest. State any variables controlled for and the rationale for controlling for them.	STORMS	In causal inference, this item refers to the assumptions required to draw causal inferences from observational data. See Vujkovic‐Cvijin, I., Sklar, J., Jiang, L. et al. Host variables confound gut microbiota studies of human disease. Nature 587, 448–454 (2020). 10.1038/s41586‐020‐2881‐9 for more details on confounding in observational microbiome studies. For example, hypothesized confounders may be controlled for by multivariable adjustment. Consider using a directed acyclic graph (DAG) to describe your causal model and justify any variables controlled for. DAGs can be made using www.dagitty.net.	No	Partial	No	Not reported	No	Not reported	No	Not reported	No	Partial
6.1	Selection bias	Discuss potential for selection or survival bias.	STORMS	Selection bias can occur when some members of the target study population are more likely to be included in the study/final analytic sample than others. Some examples include survival bias (where part of the target study population is more likely to die before they can be studied), convenience sampling (where members of the target study population are not selected at random), and loss to follow‐up (when the probability of dropping out is related to one of the things being studied).	No	Not reported	No	Not reported	Yes	Material and methods—bioinformatic analyses	No	Not reported	No	Partial
7.0	Bioinformatic and statistical methods	Describe any transformations to quantitative variables used in analyses (e.g., use of percentages instead of counts, normalization, rarefaction, and categorization).	STORMS	If a variable is analyzed using different transformations, state rationale for the transformation and for each analyses which version of the variable is used. For complex or multistep transformations, provide enumerated instructions for reproducing them.	Yes	Methods—statistical analysis	Yes	Methods—DNA purification and microbiota analysis	Yes	Material and methods—16S RNA gene sequencing	No	Partial	Yes	Methods
7.1	Quality Control	Describe any methods to identify or filter low‐quality reads or samples.	MIxS: sequence quality check	If samples were excluded based on quality or read depth, list the criteria used, the number of samples excluded, and the final sample size after quality control.	Yes	Methods—sequencing and analysis of 16S ribosomal RNA gene sequences	Yes	Methods—DNA purification and microbiota analysis	Yes	Material and methods—16S RNA gene sequencing	No	Partial	Yes	Methods
7.2	Sequence analysis	Describe any taxonomic, functional profiling, or other sequence analysis performed.	MIxS: feature prediction; similarity search method		Yes	Methods—sequencing and analysis of 16S ribosomal RNA gene sequences	Yes	Methods—DNA purification and microbiota analysis	Yes	Material and methods—bioinformatic analyses	Yes	Materials and methods—microbiota sequencing and taxonomy assignment	Yes	Methods
7.3	Statistical methods	Describe all statistical methods.	Modified STROBE	Describe any statistical tests used, exploratory data analysis performed, dimension reduction methods/unsupervised analysis, α/β metrics, and/or methods for adjusting for measurement bias. If multiple statistical methods are possible, discuss why the methods used were selected. If a multiple‐hypothesis testing correction method was used, describe the type of correction used. State which taxonomic levels are analyzed.	Yes	Methods—statistical analysis	Yes	Methods—statistical analysis	Yes	Material and methods—statistical analysis	Yes	Materials and methods—statistical analysis	Yes	Methods—statistical and machine‐learning methods
7.4	Longitudinal analysis	If the study is longitudinal, include a section that explicitly states what analysis methods were used (if any) to account for the grouping of measurements by individual or patterns over time.	STORMS		Yes	Methods—statistical analysis	NA		Yes	Material and methods—sample collection	NA		Yes	Methods—statistical and machine‐learning methods
7.5	Subgroup analysis	Describe any methods used to examine subgroups and interactions.	STROBE		Yes	Methods—statistical analysis	Yes	Methods—statistical analysis	No	Not reported	No	Partial	No	Not reported
7.6	Missing data	Explain how missing data were addressed.	STROBE	“Missing data” refers to participant measurements such as covariates, exposures, outcomes, or time points that should have been collected but were not, not to zeros in taxonomic abundance tables or data points not applicable to that observation.	No	Not reported	No	Not reported	No	Not reported	No	Not reported	No	Partial
7.7	Sensitivity analyses	Describe any sensitivity analyses.	STROBE		No	Not reported	No	Not reported	No	Not reported	No	Not reported	No	Partial
7.8	Findings	State criteria used to select findings for reporting.	STORMS	For example, false discovery rate with total number of tests, effect size threshold, significance threshold, and microbes of interest.	No	Not reported	No	Not reported	No	Not reported	No	Not reported	No	Partial
7.9	Software	Cite all software (including read mapping software) and databases (including any used for taxonomic reference or annotating amplicons, if applicable) used. Include version numbers.	Modified STREGA	Installed packages, add‐ons or libraries should be stated and cited in addition to the software used. All parameters employed that differ from the default of that software/version should be provided. This is in addition to, not a replacement for, the publishing of code as outlined in the section Reproducible Research.	Yes	Methods—sequencing and analysis of 16S ribosomal RNA gene sequences	Yes	Methods—DNA purification and microbiota analysis	Yes	Material and methods—statistical analysis	Yes	Materials and methods—microbiota sequencing and taxonomy assignment	Yes	Methods
8.0	Reproducible research	Make a statement about whether and how others can reproduce the reported analysis.	STORMS	Any protected information that has been excluded or provided under controlled access should be listed along with any relevant data access procedures. “On request from authors” is not sufficiently detailed; formal data access procedures and conditions should be defined. If data are unavailable, state so clearly. Consider using a specialized rubric for reproducible research (such as https://mbio.asm.org/content/9/3/e00525-18.short). Consider preregistering the study protocol (such as on osf.io or https://plos.org/open-science/preregistration/ ).	No	Not reported	No	Not reported	No	Not reported	No	Not reported	No	Partial
8.1	Raw data access	State where raw data may be accessed, including demultiplexing information.	STORMS	Robust, long‐term databases such as those hosted by NCBI and EBI are preferred. If using a private repository, provide a rationale.	No	Not reported	No	Not reported	No	Not reported	Yes	Materials and methods—microbiota sequencing and taxonomy assignment	No	Partial
8.2	Processed data access	State where processed data may be accessed.	STORMS	Unfiltered data should be provided. Robust, long‐term databases such as those hosted by NCBI and EBI‐EMBL are preferred. Repositories like zenodo (https://zenodo.org/) or publisso (https://www.publisso.de/en/working-for-you/doi-service/) can be used to provide a DOI and long‐term storage for processed data sets, even those which cannot be published openly.	No	Not reported	No	Not reported	No	Not reported	No	Not reported	No	Partial
8.3	Participant data access	State where individual participant data, such as demographics and other covariates, may be accessed, and how they can be matched to the microbiome data.	STORMS	If recategorized, transformed, or otherwise derived variables were used in the analysis, these variables or code for deriving them should be provided. Examples of how participant data can be matched to microbiome data are: using the same set of anonymized identifiers, or using different anonymized identifiers but providing a map. The provided data should be sufficient to independently replicate the current analysis.	No	Not reported	No	Not reported	No	Not reported	No	Not reported	No	Partial
8.4	Source code access	State where code may be accessed.	STORMS	If a standard or formalized workflow was employed, reference it here.	No	Not reported	No	Not reported	No	Not reported	No	Not reported	No	Partial
8.5	Full results	Provide full results of all analyses, in computer‐readable format, in Supplementary materials.	STORMS	For example, any fold‐changes, *p* values, or FDR values are calculated, provided as a spreadsheet. Use a machine‐readable, plain‐text format, such as csv or tsv.	No	Not reported	No	Not reported	No	Not reported	No	Not reported	No	Not reported
*Results*														
9.0	Descriptive data	Give characteristics of study participants (e.g., dietary, demographic, clinical, and social) and information on exposures and potential confounders.	STROBE	Typically reported in a table included in the paper or as a Supplementary table. Indicate the number of participants with missing data for each variable of interest. This includes environmental and lifestyle factors that may affect the relationship between the microbiome and the condition of interest. Participant diet and medication use should be summarized, if known. At a minimum, the age and sex of all participants should be summarized.	Yes	Results	Yes	Results	Yes	Results	Yes	Results	Yes	Results
10.0	Microbiome data	Report descriptive findings for microbiome analyses with all applicable outcomes and covariates.	STORMS	This includes measures of diversity as well as relative abundances. These descriptive findings should be reported both for the sample overall and for individual groups.	Yes	Results	Yes	Results	Yes	Results	Yes	Results	Yes	Results
10.1	Taxonomy	Identify taxonomy using standardized taxon classifications that are sufficient to uniquely identify taxa.	STORMS	If not using the full taxonomic hierarchy, make sure it is clear whether the names stated are species, genus, family, and so forth. Italicize genus/species pairs. Consult journal guidelines or standardized references on taxonomic nomenclature. For instance, https://wwwnc.cdc.gov/eid/page/scientific-nomenclature	Yes	Results	Yes	Results	Yes	Results	Yes	Results	Yes	Results
10.2	Differential abundance	Report results of differential abundance analysis by the variable of interest and (if applicable) by time, clearly indicating the direction of change and total number of taxa tested.	STORMS	If there are more than two groups, include omnibus (multigroup) test results if applicable to the research question. If applicable, reported effect sizes should include a measure of uncertainty, such as the confidence interval.	Yes	Results	Yes	Results	No	Not reported	Yes	Results	No	Partial
10.3	Other data types	Report other data analyzed—for example, metabolic function, functional potential, MAG assembly, and RNAseq.	STORMS		No	None described	No	Not reported	No	Not reported	No	Not reported	No	Not reported
10.4	Other statistical analysis	Report any statistical data analysis not covered above.	STORMS	This could include subgroup analysis, sensitivity analyses, and cluster analysis. Visualizations should be easily interpretable and colorblind‐friendly. The caption and/or main text should provide a detailed description of visualizations for visually‐impaired readers.	Yes	Results	Yes	Results	Yes	Results	Yes	Results	Yes	Results
*Discussion*														
11.0	Key results	Summarize key results with reference to study objectives	STROBE		Yes	Discussion	Yes	Discussion	Yes	Discussion	Yes	Discussion	Yes	Discussion
12.0	Interpretation	Give a cautious overall interpretation of results considering objectives, limitations, multiplicity of analyses, results from similar studies, and other relevant evidence.	STROBE	Define or clarify any subjective terms such as “dominant,” “dysbiosis,” and similar words used in the interpretation of results. When interpreting the findings, consider how the interpretation of the findings may be summarized or quoted for the general public, such as in press releases or news articles. If causal language is used in the interpretation (such as “alters,” “affects,” “results in,” “causes,” or “impacts”), assumptions made for causal inference should be explicitly stated as part of 6.0 and 13.0. Distinguish between function potential (i.e., inferred from metagenomics) and observed activity (i.e., metatranscriptomic, metabolomic, proteomic) if discussing microbial function.	Yes	Discussion	Yes	Discussion	Yes	Discussion	Yes	Discussion	No	Partial
13.0	Limitations	Discuss limitations of the study, taking into account sources of potential bias or imprecision.	STROBE	Also consider limitations resulting from the methods (especially novel methods), the study design, and the sample size.	Yes	Discussion	Yes	Discussion	Yes	Discussion	Yes	Discussion	No	Partial
13.1	Bias	Discuss any potential for bias to influence study findings.	STORMS	May include sampling method, representativeness of the study participants, or potential confounding factors.	Yes	Discussion	Yes	Discussion	Yes	Discussion	No	Partial	No	Partial
13.2	Generalizability	Discuss the generalizability (external validity) of the study results	STROBE	To what populations or other settings do you expect the conclusions to generalize?	Yes	Discussion	Yes	Discussion	Yes	Discussion	No	Not reported	No	Partial
14.0	Ongoing/future work	Describe potential future research or ongoing research based on the study's findings.	STORMS		Yes	Discussion	Yes	Discussion	Yes	Discussion	Yes	Discussion	Yes	Discussion
*Other information*														
15.0	Funding	Give the source of funding and the role of the funders for the present study and, if applicable, for the original study on which the present article is based	STROBE		Yes	Notes	Yes	Acknowledgments	No	Funding statement	Yes	Funding	Yes	Funding
15.1	Acknowledgments	Include acknowledgments of those who contributed to the research but did not meet criteria for authorship.	STORMS	For general guidelines on authorship, see http://www.icmje.org and https://www.elsevier.com/authors/journal-authors/policies-and-ethics/credit-author-statement	Yes	Notes	Yes	Acknowledgments	Yes	Acknowledgments	NA		NA	
15.2	Conflicts of Interest	Include a conflicts of interest statement.	STORMS		Yes	Notes	Yes	Acknowledgments	No	Statement	Yes	Conflicts of interest	Yes	Transparency declaration
16.0	Supplements	Indicate where supplements may be accessed and what materials they contain.	STORMS		Yes	Supplementary data	Yes	Supplementary data	Yes	Supporting Information	Yes	Supplementary materials	Yes	Appendix A Supplementary data
17.0	Supplementary data	Provide Supplementary data files of results for all taxa and all outcome variables analyzed. Indicate the taxonomic level of all taxa.	STORMS	Depending on the analysis performed, examples of the supplemental results included could be mean relative abundance, differential abundance, raw *p* value, multiple hypothesis testing‐adjusted *p* values, and standard error. All discussed taxa should include the taxonomic level (e.g., class, order, and genus).	No	Partial	No	Partial	Yes	Supporting Information	Yes	Supplementary materials	No	Partial
					43		39		42		29		35	
*Coverage*					62.31%		56.52%		60.90%		42.02%		50.72%	

Abbreviations: mRNA, messenger RNA; qPCR, quantitative polymerase chain reaction; rRNA, ribosomal ribonucleic acid; STORMS, Strengthening the Organization and Reporting of Microbiome Studies.

**Table A10b:** STORMS checklist, part 2.

Version	1.03				Garcia‐Garcia, 03/2023		Baek, 04/2023		Prevel, 04/2023		Ling, 09/2025		Park, 09/2025	
Number	Item	Recommendation	Item source	Additional guidance	Yes/No/NA	Comments or location in manuscript	Yes/No/NA	Comments or location in manuscript	Yes/No/NA	Comments or location in manuscript	Yes/No/NA	Comments or location in manuscript	Yes/No/NA	Comments or location in manuscript
*Abstract*														
1.0	Structured or unstructured abstract	Abstract should include information on background, methods, results, and conclusions in a structured or unstructured format.	STORMS		Yes	Abstract	Yes	Abstract	No	Abstract	Yes	Abstract	Yes	Abstract
1.1	Study design	State study design in abstract.	STORMS	See 3.0 for additional information on study design.	No	Partial	No	Partial	No	Not reported	Yes	Abstract—methods and results	No	Not reported
1.2	Sequencing methods	State the strategy used for metagenomic classification.	STORMS	For example, targeted 16S by qPCR or sequencing, shotgun metagenomics, metatranscriptomics, and so forth.	No	Partial	Yes	Abstract—methods	No	Not reported	Yes	Abstract—methods and results	Yes	Abstract—methods
1.3	Specimens	Describe body site(s) studied.	STORMS		Yes	Abstract	Yes	Abstract—methods	Yes	Abstract	No	Partial	Yes	Abstract—methods
*Introduction*														
2.0	Background and Rationale	Summarize the underlying background, scientific evidence, or theory driving the current hypothesis as well as the study objectives.	STORMS		Yes	Introduction	Yes	Introduction	Yes	Background	Yes	Introduction	Yes	Introduction
2.1	Hypotheses	State the prespecified hypothesis. If the study is exploratory, state any prespecified study objectives.	STORMS		Yes	Introduction	Yes	Introduction	Yes	Background	Yes	Introduction	Yes	Introduction
*Methods*														
3.0	Study Design	Describe the study design.	STORMS	Observational (case–control, cohort, cross‐sectional survey, etc.) or Experimental (Randomized controlled trial, nonrandomized controlled trial, etc.). For a brief description of common study designs, see: DOI: 10.11613/BM.2014.022 If applicable, describe any blinding (e.g., single or double‐blinding) used in the course of the study.	Yes	Materials and methods—study design	Yes	Materials and methods—study design and eligible patients	Yes	Methods—patient inclusion and data collection	Yes	Methods—participants' enrollment	Yes	Methods—population
3.1	Participants	State what the population of interest is, and the method by which participants are sampled from that population. Include relevant information on physiological state of the subjects or stage in the life history of disease under study when participants were sampled.	STORMS	Examples of the population of interest could be: adults with no chronic health conditions, adults with type II diabetes, newborns, and so forth. This is the total population to whom the study is hoped to be generalizable to. The sampling method describes how potential participants were selected from that population. If the participants are from a substudy of a larger study, provide a brief description of that study and cite that study. Clearly state how cases and controls are defined. An example of relevant physiological state might be pre/post menopausal for a vaginal microbiome study; examples of stage in the life history of disease could be whether specimens were collected during active or dormant disease, or before or after treatment.	Yes	Materials and methods—patient population	Yes	Materials and methods—study design and eligible patients	Yes	Methods—patient inclusion and data collection	Yes	Methods—participants' enrollment	Yes	Methods—population
3.2	Geographic location	State the geographic region(s) where participants were sampled from.	MIxS: geographic location (country and/or sea, region)	Geographic coordinates can be reported to prevent potential ambiguities if necessary.	Yes	Materials and methods—patient population	Yes	Materials and methods—study design and eligible patients	Yes	Methods—patient inclusion and data collection	Yes	Methods—participants' enrollment	No	Not reported
3.3	Relevant dates	State the start and end dates for recruitment, follow‐up, and data collection.	STORMS	Recruitment is the period in which participants are recruited for the study. In longitudinal studies, follow‐up is the date range in which participants are asked to complete a specific assessment. Finally, data collection is the period during which data are collected from participants, from initial recruitment through all follow‐ups.	Yes	Materials and methods—patient population	Yes	Materials and methods—study design and eligible patients	Yes	Methods—patient inclusion and data collection	Yes	Methods—participants' enrollment	No	Not reported
3.4	Eligibility criteria	List any criteria for inclusion and exclusion of recruited participants.	Modified STROBE	Among potential recruited participants, how were some chosen and others not? This could include criteria, such as sex, diet, age, health status, or BMI. If there is a primary and validation sample, describe the inclusion/exclusion criteria for each.	Yes	Materials and methods—patient population	Yes	Materials and methods—study design and eligible patients	No	Not reported	Yes	Methods—participants' enrollment	Yes	Methods—population
3.5	Antibiotics Usage	List what is known about antibiotic usage before or during sample collection.	STORMS	If participants were excluded due to current or recent antibiotic usage, state this here. Other factors (e.g., proton pump inhibitors, probiotics, etc.) that may influence the microbiome should also be described as well.	No	Not reported	Yes	Materials and methods—data collection and definitions	Yes	Methods—patient inclusion and data collection	Yes	Methods—participants' enrollment	Yes	Methods—population
3.6	Analytic sample size	Explain how the final analytic sample size was calculated, including the number of cases and controls if relevant, and reasons for dropout at each stage of the study. This should include the number of individuals in whom microbiome sequencing was attempted and the number in whom microbiome sequencing was successful.	STORMS	Consider use of a flow diagram (see template at https://stormsmicrobiome.org/figures). Also state sample size in abstract. If power analysis was used to calculate sample size, describe those calculations.	No	Not reported	No	Not reported	No	Not reported	Yes	Methods—participants' enrollment	No	Not reported
3.7	Longitudinal Studies	For longitudinal studies, state how many follow‐ups were conducted, describe the sample size at follow‐up by group or condition, and discuss any loss to follow‐up.	STORMS	If there is loss to follow‐up, discuss the likelihood that drop‐out is associated with exposures, treatments, or outcomes of interest.	NA		NA		NA		NA		Yes	Methods
3.8	Matching	For matched studies, give matching criteria.	Modified STROBE	“Matched” refers to matching between comparable study participants as cases and controls or exposed/unexposed. Indicate whether participants were individual or frequency matched and in what ratio were they matched (e.g., 1 case to 1 control).	No	Not reported	No	Not reported	Yes	Noncarriers matched on sex and age ±5 years	Yes	healthy controls matched on gender and age	No	Not reported
3.9	Ethics	State the name of the institutional review board that approved the study and protocols, protocol number, and date of approval, and procedures for obtaining informed consent from participants.	STORMS		Yes	Materials and methods—ethics statement	Yes	Materials and methods—study design and eligible patients	Yes	Methods—ethics	Yes	Methods—participants' enrollment	No	Not reported
4.0	Laboratory methods	State the laboratory/center where laboratory work was done.	STORMS	Provide a reference to complete lab protocols if previously published elsewhere, such as on protocols.io. Note any modifications of lab protocols and the reason for protocol modifications.	Yes	Materials and methods—microbial DNA extraction, library preparation, and next‐generation sequencing	Yes	Materials and methods—microbiome analysis	Yes	Methods—sample collection and preparation for microbiota analysis	Yes	Methods—library preparation and sequencing	No	Not reported
4.1	Specimen collection	State the body site(s) sampled from and how specimens were collected.	MIxS: sample collection device or method; host body site	Use terms from the Uber‐anatomy Ontology (https://www.ebi.ac.uk/ols/ontologies/uberon) to describe body sites in a standardized format.	Yes	Materials and methods—study design	Yes	Materials and methods—microbiome analysis	No	Not reported	Yes	Methods—sample collection	Yes	Methods—samples
4.2	Shipping	Describe how samples were stored and shipped to the laboratory.	STORMS	Include length of time from collection to receipt by the lab and if temperature control was used during shipping.	No	Not reported	No	Not reported	Yes	Methods—sample collection and preparation for microbiota analysis	No	Methods—sample collection	No	Not reported
4.3	Storage	Describe how the laboratory stored samples, including the time between collection and storage, and any preservation buffers or refrigeration used.	STORMS	State where each procedure or lot of samples was done if Not all in the same place. Include reagent/lot/catalog #s for storage buffers.	No	Not reported	Yes	Materials and methods	Yes	Methods—sample collection and preparation for microbiota analysis	Yes	Methods—sample collection	Yes	Methods—samples
4.4	DNA extraction	Provide DNA extraction method, including kit and version if relevant.	MIxS: nucleic acid extraction	If any DNA quantification methods were used prior to DNA amplification or at the pooling step of library preparation, state so here.	Yes	Materials and methods—microbial DNA extraction, library preparation, and next‐generation sequencing	Yes	Materials and methods—microbiome analysis	Yes	Methods—sample collection and preparation for microbiota analysis	Yes	Methods—library preparation and sequencing	Yes	Methods—16S rRNA gene sequencing
4.5	Human DNA sequence depletion or microbial DNA enrichment	Describe whether human DNA sequence depletion or enrichment of microbial or viral DNA was performed.	STORMS		No	Not reported	No	Partial	No	Not reported	No	Not reported	No	Not reported
4.6	Primer selection	Provide primer selection and DNA amplification methods as well as variable region sequenced (if applicable).	MIxS: PCR primers		Yes	Materials and methods—microbial DNA extraction, library preparation, and next‐generation sequencing	Yes	Materials and methods—microbiome analysis	Yes	Methods—sample collection and preparation for microbiota analysis	Yes	Methods—library preparation and sequencing	Yes	Methods—16S rRNA gene sequencing
4.7	Positive Controls	Describe any positive controls (mock communities) if used.	STORMS	If used, should be deposited under guidance provided in the 8.X items.	No	Not reported	No	Partial	Yes	Methods—bioinformatic analysis	Yes	Methods	Yes	Methods—16S rRNA gene sequencing
4.8	Negative Controls	Describe any negative controls if used.	STORMS	If used, should be deposited under guidance provided in the 8.X items.	No	Not reported	No	Partial	Yes	Methods—bioinformatic analysis	Yes	Methods—library preparation and sequencing	Yes	Methods—16S rRNA gene sequencing
4.9	Contaminant mitigation and identification	Provide any laboratory or computational methods used to control for or identify microbiome contamination from the environment, reagents, or laboratory.	STORMS	Includes filtering of reagents and other steps to minimize contamination. It is relevant to state whether the specimens of interest have low microbial load, which makes contamination especially relevant.	No	Partial	No	Partial	Yes	Methods—bioinformatic analysis	Yes	Methods—library preparation and sequencing	Yes	Methods—16S rRNA gene sequencing
4.10	Replication	Describe any biological or technical replicates included in the sequencing, including which steps were replicated between them.	STORMS	Replication may be biological (redundant biological specimens) or technical (aliquots taken at different stages of analysis) and used in extraction, sequencing, preprocessing, and/or data analysis.	No	Not reported	No	Partial	No	Not reported	Yes	Methods—library preparation and sequencing	No	Not reported
4.11	Sequencing strategy	Major divisions of strategy, such as shotgun or amplicon sequencing.	MIxS: sequencing method	For amplicon sequencing (e.g., 16S variable region), state the region selected. State the model of the sequencer used.	Yes	Materials and methods—microbial DNA extraction, library preparation, and next‐generation sequencing	Yes	Materials and methods—microbiome analysis	Yes	Methods—sample collection and preparation for microbiota analysis	Yes	Methods—library preparation and sequencing	Yes	Methods—16S rRNA gene sequencing
4.12	Sequencing methods	State whether experimental quantification was used (QMP/cell count based, spike‐in based) or whether relative abundance methods were applied.	STORMS	These include read length, sequencing depth per sample (average and minimum), whether reads are paired, and other parameters.	No	Partial	No	Partial	No	Methods—bioinformatic analysis	Yes	Methods—library preparation and sequencing	Yes	Methods—16S rRNA gene sequencing
4.13	Batch effects	Detail any blocking or randomization used in study design to avoid confounding of batches with exposures or outcomes. Discuss any likely sources of batch effects, if known.	STORMS	Sources of batch effects include sample collection, storage, library preparation, and sequencing, and are commonly unavoidable in all but the smallest of studies.	No	Not reported	No	Partial	No	Not reported	No	Not reported	No	Not reported
4.14	Metatranscriptomics	Detail whether any mRNA enrichment was performed and whether/how retrotranscription was performed prior to sequencing. Provide a size range of isolated transcripts. Describe whether the sequencing library was stranded or not. Provide details on sequencing methods and platforms.	STORMS	Provide details on any internal standards that may have been used, as well as parameters and versions of any software or databases used.	NA		NA		NA		NA		NA	
4.15	Metaproteomics	Detail which protease was used for digestion. Provide details on proteomic methods and platforms (e.g., LC‐MS/MS, instrument type, column type, mass range, resolution, scan speed, maximum injection time, isolation window, normalized collision energy, and resolution).	STORMS	Provide details on any internal standards that may have been used, as well as parameters and versions of any software or databases used.	NA		NA		NA		NA		NA	
4.16	Metabolomics	Specify the analytic method used (such as nuclear magnetic resonance spectroscopy or mass spectrometry). For mass spectrometry, provide details on which fractions were obtained (polar and/or nonpolar) and how these were analyzed. Provide details on metabolomics methods and platforms (e.g., derivatization, instrument type, injection type, column type, and instrument settings).	STORMS	Provide details on any internal standards that may have been used, as well as parameters and versions of any software or databases used.	NA		Yes	Materials and methods—quantitative measurement of short‐chain fatty acids	NA		NA		NA	
5.0	Data sources/measurement	For each nonmicrobiome variable, including the health condition, intervention, or other variable of interest, state how it was defined, how it was measured or collected, and any transformations applied to the variable prior to analysis.	MIxS: host disease status	State any sources of potential bias in measurements, for example, multiple interviewers or measurement instruments, and whether these potential biases were assessed or accounted for in the study design. Use terms from a standardized ontology, such as the Experimental Factor Ontology (https://www.ebi.ac.uk/efo/), to describe variables of interest in a standardized format.	Yes	Materials and methods—study design	Yes	Materials and methods—data collection and definitions	Yes	Methods—patient inclusion and data collection	Yes	Methods—systematic inflammatory cytokines analysis	Yes	Methods
6.0	Research design for causal inference	Discuss any potential for confounding by variables that may influence both the outcome and exposure of interest. State any variables controlled for and the rationale for controlling for them.	STORMS	For causal inference, this item refers to describing the assumptions that would be required to draw causal inferences from observational data. See Vujkovic‐Cvijin, I., Sklar, J., Jiang, L., et al. Host variables confound gut microbiota studies of human disease. Nature 587, 448–454 (2020). 10.1038/s41586‐020‐2881‐9 for more details on confounding in observational microbiome studies. For example, hypothesized confounders may be controlled for by multivariable adjustment. Consider using a directed acyclic graph (DAG) to describe your causal model and justify any variables controlled for. DAGs can be made using www.dagitty.net.	No	Not reported	No	Partial	No	Not reported	No	Not reported	No	Not reported
6.1	Selection bias	Discuss potential for selection or survival bias.	STORMS	Selection bias can occur when some members of the target study population are more likely to be included in the study/final analytic sample than others. Some examples include survival bias (where part of the target study population is more likely to die before they can be studied), convenience sampling (where members of the target study population are not selected at random), and loss to follow‐up (when the probability of dropping out is related to one of the things being studied).	No	Partial	No	Partial	No	Not reported	No	Not reported	No	Not reported
7.0	Bioinformatic and statistical methods	Describe any transformations to quantitative variables used in analyses (e.g., use of percentages instead of counts, normalization, rarefaction, and categorization).	STORMS	If a variable is analyzed using different transformations, state rationale for the transformation and for each analyses which version of the variable is used. In case of any complex or multistep transformations, give enumerated instructions for reproducing those transformations.	Yes		Yes	Materials and methods	Yes	Methods—bioinformatic analysis	Yes	Methods	Yes	Methods—statistical analysis
7.1	Quality Control	Describe any methods to identify or filter low‐quality reads or samples.	MIxS: sequence quality check	If samples were excluded based on quality or read depth, list the criteria used, the number of samples excluded, and the final sample size after quality control.	Yes	Materials and methods—bioinformatics and statistical analysis	Yes	Materials and methods—microbiome analysis	No	Not reported	Yes	Methods—data processing	Yes	Methods—16S rRNA gene sequencing
7.2	Sequence analysis	Describe any taxonomic, functional profiling, or other sequence analysis performed.	MIxS: feature prediction; similarity search method		Yes	Materials and methods—bioinformatics and statistical analysis	Yes	Materials and methods—microbiome analysis	Yes	Methods—bioinformatic analysis	Yes	Methods—data processing	Yes	Methods—16S rRNA gene sequencing
7.3	Statistical methods	Describe all statistical methods.	Modified STROBE	Describe any statistical tests used, exploratory data analysis performed, dimension reduction methods/unsupervised analysis, α/β metrics, and/or methods for adjusting for measurement bias. If multiple statistical methods are possible, discuss why the methods used were selected. If a multiple‐hypothesis testing correction method was used, describe the type of correction used. State which taxonomic levels are analyzed.	Yes	Materials and methods—bioinformatics and statistical analysis	Yes	Materials and methods—statistical analysis	Yes	Methods	Yes	Methods—statistical analysis	Yes	Methods—statistical analysis
7.4	Longitudinal analysis	If the study is longitudinal, include a section that explicitly states what analysis methods were used (if any) to account for the grouping of measurements by individual or patterns over time.	STORMS		NA		NA		NA		NA		Yes	Methods—statistical analysis
7.5	Subgroup analysis	Describe any methods used to examine subgroups and interactions.	STROBE		No	Partial	No	Partial	Yes	Methods—statistical analysis	Yes	Methods—statistical analysis	No	Not reported
7.6	Missing data	Explain how missing data were addressed.	STROBE	“Missing data” refers to participant measurements such as covariates, exposures, outcomes, or time points that should have been collected but were not, not to zeros in taxonomic abundance tables, or data points not applicable to that observation.	No	Not reported	No	Partial	No	Not reported	Yes	Methods—systematic inflammatory cytokines analysis	Yes	Methods—statistical analysis
7.7	Sensitivity analyses	Describe any sensitivity analyses.	STROBE		No	Not reported	No	Partial	No	Not reported	Yes	Methods—statistical analysis	No	Not reported
7.8	Findings	State criteria used to select findings for reporting.	STORMS	For example, false discovery rate with total number of tests, effect size threshold, significance threshold, and microbes of interest.	Yes	Materials and methods—bioinformatics and statistical analysis	Yes	Materials and methods—microbiome analysis	No	Not reported	Yes	Methods—statistical analysis	No	Not reported
7.9	Software	Cite all software (including read mapping software) and databases (including any used for taxonomic reference or annotating amplicons, if applicable) used. Include version numbers.	Modified STREGA	Installed packages, add‐ons, or libraries should be stated and cited in addition to the software used. All parameters employed that differ from the default of that software/version should be provided. This is in addition to, not a replacement for, the publishing of code as outlined in the section Reproducible Research.	Yes	Materials and methods—bioinformatics and statistical analysis	Yes	Materials and methods	Yes	Methods	Yes	Methods	Yes	Methods—16S rRNA gene sequencing
8.0	Reproducible research	Make a statement about whether and how others can reproduce the reported analysis.	STORMS	Any protected information that has been excluded or provided under controlled access should be listed along with any relevant data access procedures. “On request from authors” is not sufficiently detailed; formal data access procedures and conditions should be defined. If data are unavailable, state so clearly. Consider using a specialized rubric for reproducible research (such as https://mbio.asm.org/content/9/3/e00525-18.short ). Consider preregistering the study protocol (such as on osf.io or https://plos.org/open-science/preregistration/ ).	No	Partial	No	Partial	No	Not reported	No	Not reported	No	Not reported
8.1	Raw data access	State where raw data may be accessed, including demultiplexing information.	STORMS	Robust, long‐term databases such as those hosted by NCBI and EBI are preferred. If using a private repository, provide a rationale.	Yes	Materials and methods—microbial DNA extraction, library preparation, and next‐generation sequencing	Yes	Not reported	Yes	Methods—bioinformatics	Yes	Methods—accession number	Yes	Methods—16S rRNA gene sequencing
8.2	Processed data access	State where processed data may be accessed.	STORMS	Unfiltered data should be provided. Robust, long‐term databases such as those hosted by NCBI and EBI‐EMBL are preferred. Repositories like zenodo (https://zenodo.org/) or publisso (https://www.publisso.de/en/working-for-you/doi-service/) can be used to provide a DOI and long‐term storage for processed data sets, even those which cannot be published openly.	No	Partial	No	Not reported	No	Not reported	No	Not reported	No	Not reported
8.3	Participant data access	State where individual participant data, such as demographics and other covariates, may be accessed, and how they can be matched to the microbiome data.	STORMS	If recategorized, transformed, or otherwise derived variables were used in the analysis, these variables or code for deriving them should be provided. Examples of how participant data can be matched to microbiome data are: using the same set of anonymized identifiers, or using different anonymized identifiers but providing a map. The provided data should be sufficient to independently replicate the current analysis.	No	Not reported	No	Not reported	No	Not reported	No	Not reported	No	Not reported
8.4	Source code access	State where code may be accessed.	STORMS	If a standard or formalized workflow was employed, reference it here.	No	Not reported	No	Not reported	Yes	Not reported	No	Not reported	No	Not reported
8.5	Full results	Provide full results of all analyses, in computer‐readable format, in the Supplementary materials.	STORMS	For example, any fold‐changes, *p* values, or FDR values calculated, provided as a spreadsheet. Use a machine‐readable, plain‐text format, such as csv or tsv.	No	Partial	No	Not reported	No	Not reported	No	Not reported	No	Not reported
*Results*														
9.0	Descriptive data	Give characteristics of study participants (e.g., dietary, demographic, clinical, and social) and information on exposures and potential confounders.	STROBE	Typically reported in a table included in the paper or as a Supplementary table. Indicate the number of participants with missing data for each variable of interest. This includes environmental and lifestyle factors that may affect the relationship between the microbiome and the condition of interest. Participant diet and medication use should be summarized, if known. At minimum, age and sex of all participants should be summarized.	Yes	Results	Yes	Results	Yes	Results	Yes	Results	Yes	Results
10.0	Microbiome data	Report descriptive findings for microbiome analyses with all applicable outcomes and covariates.	STORMS	This includes measures of diversity as well as relative abundances. These descriptive findings should be reported both for the sample overall and for individual groups.	Yes	Results	No	Partial	Yes	Results	Yes	Results	Yes	Results
10.1	Taxonomy	Identify taxonomy using standardized taxon classifications that are sufficient to uniquely identify taxa.	STORMS	If not using full taxonomic hierarchy, make sure it is clear whether names stated are species, genera, family, and so forth. Italicize genus/species pairs. Consult journal guidelines or standardized references on taxonomic nomenclature. For instance, https://wwwnc.cdc.gov/eid/page/scientific-nomenclature	Yes	Results	Yes	Results	Yes	Results	Yes	Results	Yes	Results
10.2	Differential abundance	Report results of differential abundance analysis by the variable of interest and (if applicable) by time, clearly indicating the direction of change and total number of taxa tested.	STORMS	If there are more than two groups, include omnibus (multigroup) test results if applicable to the research question. If applicable, reported effect sizes should include a measure of uncertainty, such as the confidence interval.	Yes	Results	No	Partial	Yes	Results	Yes	Results	Yes	Results
10.3	Other data types	Report other data analyzed—for example, metabolic function, functional potential, MAG assembly, and RNAseq.	STORMS		No	Not reported	No	Partial	No	Not reported	Yes	Results	Yes	Results
10.4	Other statistical analysis	Report any statistical data analysis not covered above.	STORMS	This could include subgroup analysis, sensitivity analyses, and cluster analysis. Visualizations should be easily interpretable and colorblind‐friendly. The caption and/or main text should provide a detailed description of visualizations for visually‐impaired readers.	Yes	Results	No	Partial	No	Not reported	Yes	Results	Yes	Results
*Discussion*														
11.0	Key results	Summarize key results with reference to study objectives	STROBE		Yes	Discussion	Yes	Discussion	Yes	Discussion	Yes	Discussion	Yes	Discussion
12.0	Interpretation	Give a cautious overall interpretation of results considering objectives, limitations, multiplicity of analyses, results from similar studies, and other relevant evidence.	STROBE	Define or clarify any subjective terms such as “dominant,” “dysbiosis,” and similar words used in the interpretation of results. When interpreting the findings, consider how the interpretation of the findings may be summarized or quoted for the general public, such as in press releases or news articles. If causal language is used in the interpretation (such as “alters,” “affects,” “results in,” “causes,” or “impacts”), assumptions made for causal inference should be explicitly stated as part of 6.0 and 13.0. Distinguish between function potential (i.e., inferred from metagenomics) and observed activity (i.e., metatranscriptomic, metabolomic, proteomic) if discussing microbial function.	Yes	Discussion	Yes	Discussion	Yes	Discussion	Yes	Discussion	Yes	Discussion
13.0	Limitations	Discuss limitations of the study, taking into account sources of potential bias or imprecision.	STROBE	Also consider limitations resulting from the methods (especially novel methods), the study design, and the sample size.	Yes	Discussion	Yes	Discussion	Yes	Discussion	Yes	Discussion	Yes	Discussion
13.1	Bias	Discuss any potential for bias to influence study findings.	STORMS	May include sampling method, representativeness of study participants, or potential confounding.	No	Partial	Yes	Discussion	Yes	Discussion	No	Partial	Yes	Discussion
13.2	Generalizability	Discuss the generalizability (external validity) of the study results	STROBE	To what populations or other settings do you expect the conclusions to generalize?	No	Not reported	No	Partial	No	Not reported	Yes	Discussion	Yes	Discussion
14.0	Ongoing/future work	Describe potential future research or ongoing research based on the study's findings.	STORMS		No	Not reported	No	Partial	Yes	Discussion	Yes	Discussion	Yes	Discussion
*Other information*														
15.0	Funding	Give the source of funding and the role of the funders for the present study and, if applicable, for the original study on which the present article is based	STROBE		Yes	Funding	Yes	Funding	Yes	Funding	Yes	Funding	Yes	Funding
15.1	Acknowledgments	Include acknowledgments of those who contributed to the research but did not meet criteria for authorship.	STORMS	For general guidelines on authorship, see http://www.icmje.org and https://www.elsevier.com/authors/journal-authors/policies-and-ethics/credit-author-statement	NA		NA		Yes	Acknowledgments	NA		NA	
15.2	Conflicts of Interest	Include a conflicts of interest statement.	STORMS		Yes	Conflicts of interest	Yes	Conflicts of interest	Yes	Competing interests	Yes	Ethics declarations	Yes	disclosures
16.0	Supplements	Indicate where supplements may be accessed and what materials they contain.	STORMS		No	Partial	No	Partial	Yes	Supplementary information	No	Partial	Yes	Supplementary material
17.0	Supplementary data	Provide Supplementary data files of results for all taxa and all outcome variables analyzed. Indicate the taxonomic level of all taxa.	STORMS	Depending on the analysis performed, examples of the supplemental results included could be mean relative abundance, differential abundance, raw *p* value, multiple hypothesis testing‐adjusted *p* values, and standard error. All discussed taxa should include the taxonomic level (e.g., class, order, and genus).	No	Partial	No	Partial	No	Partial	No	Partial	No	Partial
					33		35		40		49		43	
*Coverage*					**47.82%**		**50.72%**		**58%**		**71.01%**		**62.31%**	

Abbreviations: EBI, European Bioinformatics Institute; LC‐MS, liquid chromatography‐mass spectrometry; mRNA, messenger RNA; NCBI, National Center for Biotechnology Information; QMP, quantitative microbiome profiling; qPCR, quantitative polymerase chain reaction; rRNA, ribosomal ribonucleic acid; STORMS, Strengthening the Organization and Reporting of Microbiome Studies.
